# Larval sites of the mosquito *Aedes aegypti formosus* in forest and domestic habitats in Africa and the potential association with oviposition evolution

**DOI:** 10.1002/ece3.8332

**Published:** 2021-11-09

**Authors:** Siyang Xia, Hany K. M. Dweck, Joel Lutomiah, Rosemary Sang, Carolyn S. McBride, Noah H. Rose, Diego Ayala, Jeffrey R. Powell

**Affiliations:** ^1^ Department of Ecology and Evolutionary Biology Yale University New Haven Connecticut USA; ^2^ Department of Molecular, Cellular and Developmental Biology Yale University New Haven Connecticut USA; ^3^ Arbovirus/Viral Hemorrhagic Fever Laboratory Center for Virus Research Kenya Medical Research Institute Nairobi Kenya; ^4^ Department of Ecology & Evolutionary Biology Princeton University Princeton New Jersey USA; ^5^ Princeton Neuroscience Institute Princeton University Princeton New Jersey USA; ^6^ UMR MIVEGEC Univ. Montpellier CNRS IRD Montpellier France; ^7^ CIRMF Franceville Gabon

**Keywords:** *Aedes aegypti*, Africa, forest and domestic habitat, larval site, microenvironment, oviposition preference

## Abstract

Adaptations to anthropogenic domestic habitats contribute to the success of the mosquito *Aedes aegypti* as a major global vector of several arboviral diseases. The species inhabited African forests before expanding into domestic habitats and spreading to other continents. Despite a well‐studied evolutionary history, how this species initially moved into human settlements in Africa remains unclear. During this initial habitat transition, African *Ae. aegypti* switched their larval sites from natural water containers like tree holes to artificial containers like clay pots. Little is known about how these natural versus artificial containers differ in their characteristics. Filling this knowledge gap could provide valuable information for studying the evolution of *Ae. aegypti* associated with larval habitat changes. As an initial effort, in this study, we characterized the microenvironments of *Ae. aegypti* larval sites in forest and domestic habitats in two African localities: La Lopé, Gabon, and Rabai, Kenya. Specifically, we measured the physical characteristics, microbial density, bacterial composition, and volatile chemical profiles of multiple larval sites. In both localities, comparisons between natural containers in the forests and artificial containers in the villages revealed significantly different microenvironments. We next examined whether the between‐habitat differences in larval site microenvironments lead to differences in oviposition, a key behavior affecting larval distribution. Forest *Ae. aegypti* readily accepted the artificial containers we placed in the forests. Laboratory choice experiments also did not find distinct oviposition preferences between forest and village *Ae. aegypti* colonies. These results suggested that African *Ae. aegypti* are likely generalists in their larval site choices. This flexibility to accept various containers with a wide range of physical, microbial, and chemical conditions might allow *Ae. aegypti* to use human‐stored water as fallback larval sites during dry seasons, which is hypothesized to have initiated the domestic evolution of *Ae. aegypti*.

## Graphical abstract

This study characterized *Aedes aegypti* larval sites in forest versus domestic habitats in La Lope, Gabon, and Rabai, Kenya. The results suggested that larval sites in different habitats have very different microenvironments. However, behavioral experiments did not find strong evidence of ovipositional difference, suggesting that African *Ae. aegypti* could be a generalist in oviposition and larval site choices.

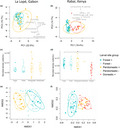

## 1 INTRODUCTION

Species that evolve in natural habitats respond differently to anthropogenic habitats, such as villages and urban areas (McKinney, 2008; Otto, 2018; Szulkin et al., 2020). While most species are unable to make this transition, a handful of species have successfully exploited these novel human environments (Johnson & Munshi‐South, 2017). Among them, one of the most notorious examples is the yellow fever mosquito, *Aedes aegypti*, a primary vector of several arboviral diseases that cause millions of infections each year, including yellow fever, dengue, chikungunya, and Zika (Aubry et al., 2020; World Health Organization, 2014). The species' high efficacy at transmitting diseases stems partly from their adaptation to human‐made domestic habitats, assuring close contact with humans (Carvalho & Moreira, 2017; Fontenille & Powell, 2020).

The evolution of *Ae. aegypti* has been strongly linked to human activities in the last few thousand years (Powell et al., 2018; Powell & Tabachnick, 2013). Genetic data suggest that *Ae. aegypti* likely originated on southwestern Indian Ocean islands and moved to continental Africa around 85,000 years ago, before spreading across the continent in tropical forests (Soghigian et al., 2020). Approximately 5000–10,000 years ago, this species expanded from their natural forest habitats into human settlements (Crawford et al., 2017; Kotsakiozi et al., 2018). They later spread to the rest of the world, likely a few hundred years ago from West Africa and facilitated by the slave trade (Brown et al., 2014; Gloria‐Soria et al., 2016; Powell & Tabachnick, 2013). Extant mosquitoes in and out of Africa are genetically distinct (Gloria‐Soria et al., 2016, but see exceptions in Kotsakiozi et al., 2018 and Rose et al., 2020) and roughly correspond to the two subspecies: *Ae. aegypti formosus* (Aaf) and *Ae. aegypti aegypti* (Aaa). Complexities exist in this subspecies definition (Powell & Tabachnick, 2013), but in this paper, we refer to them simply based on their geographic range (in or out of Africa). Non‐African Aaa is mainly associated with human dwellings (McBride, 2016; Powell & Tabachnick, 2013), except for a few populations in the Caribbean and Argentina that use ancestral natural larval habitats (Chadee et al., 1998; Mangudo et al., 2015). On the other hand, African Aaf inhabits both forest and domestic habitats (Kotsakiozi et al., 2018; Paupy et al., 2014; Sylla et al., 2009), with the latter likely representing an intermediate step between the ancestral forest‐living Aaf and the human‐associated Aaa outside of Africa. In this study, we focus primarily on Aaf in Africa with a comparison of forest‐living and domestic‐living populations.

Despite a well‐characterized evolutionary path of *Ae. aegypti* deduced from genetic data, how this species initially moved into domestic habitats in Africa is not fully understood. For *Ae. aegypti*, the transition from the ancestral forest habitat to human settlement involved two major behavioral changes: a preference for humans as a blood source (McBride, 2016) and using human‐made containers as larval habitats (Day, 2016). Ancestral forest‐living Aaf in Africa is a feeding generalist and bites wildlife for blood, while Aaa out of Africa prefers to bite humans (McBride, 2016; Powell et al., 2018). A recent study demonstrated that the variation in blood‐feeding preference across Africa is strongly associated with the length of dry seasons and human population density, implying that these factors were major ecological drivers for *Ae. aegypti's* domestication (Rose et al., 2020).

In comparison, larval sites are relatively understudied, especially in Africa. *Aedes aegypti* lay eggs on the inner surfaces of containers above the waterline (Christophers, 1960). Non‐African *Aaa* uses mostly artificial containers, consistent with their close association with humans (Day, 2016; Swan et al., 2018; Vezzani, 2007; Yee, 2008). In Africa, however, Aaf in the forest and domestic habitats utilize different larval sites: the former uses natural containers like rainwater‐filled tree holes and rock pools (Lounibos, 1981), while the latter is similar to Aaa in its reliance on artificial containers, such as plastic buckets, tires, and clay pots (Leahy et al., 1978; McBride et al., 2014; Ngugi et al., 2017; Petersen, 1977; Philbert & Ijumba, 2013; Wilson‐Bahun et al., 2020). Some artificial containers hold water year‐round and could provide valuable or even the only available larval habitats during the dry season when natural containers dry out. Therefore, it has been hypothesized that seeking human water storage for oviposition during the dry season likely drove Aaf to enter domestic habitats, leading to the evolution of feeding preference for humans (Petersen, 1977; Powell et al., 2018; Rose et al., 2020). Despite the presumed key role of larval habitats in the domestic adaptations of *Ae. aegypti*, little data exists on the detailed microenvironment of natural and artificial larval sites in Africa (but see Dickson et al., 2017; Onchuru et al., 2016).

Larval habitats are closely tied to female oviposition preferences, though differential larval survival may also lead to differences in larval abundance and distribution. Many environmental factors can affect female oviposition and larval development. For instance, studies on oviposition preference generally found a higher attractiveness of *Ae. aegypti* for larger container size and water volume (Bond & Fay, 1969; Harrington et al., 2008), more shading (Prado et al., 2017; Vezzani & Albicócco, 2009), and lower water salinity (Matthews et al., 2019). Conspecific larval density (Zahiri & Rau, 1998), bacterial density and composition (Arbaoui & Chua, 2014; Hazard et al., 1967; Ponnusamy et al., 2015) as well as chemical components (Afify & Galizia, 2015; Melo et al., 2019) were also important for oviposition choices. In addition, temperature and humidity could affect female reproductivity (Canyon et al., 1999; Costa et al., 2010), thus influencing oviposition. Lastly, larval development depends on temperature (Couret et al., 2014; Mohammed & Chadee, 2011) and nutrition (Merritt et al., 1992), with the latter often correlated with microbial density and composition (Souza et al., 2019). Therefore, if forest and domestic larval sites have distinct microenvironments, Aaf in the two habitats could have different oviposition preferences or larval adaptations.

To fill the knowledge gaps on Aaf larval habitats, in this study, we characterized forest and domestic larval sites in two African localities: La Lopé in Gabon and Rabai in Kenya. *Aedes aegypti* in both localities are Aaf based on their morphology and genetic variation (Kotsakiozi et al., 2018; Xia et al., 2020), but can be found in both forest and village environments. In each locality, forest and village populations showed little genetic differentiation (Xia et al., 2020), suggesting local habitat expansion instead of external introduction. While studies going back to the 1970s indicated indoor‐breeding *Ae*. *aegypti* in Rabai corresponding to Aaa (Leahy et al., 1978; McBride et al., 2014; Petersen, 1977; Tabachnick et al., 1979; Trpis & Hausermann, 1975), which were likely introduced from outside Africa (Brown et al., 2011; Gloria‐Soria et al., 2016), we no longer found this *Aaa*‐like indoor form during this study. All *Ae. aegypti* collected in Rabai in this study were Aaf (Rose et al., 2020; Xia et al., 2020).

The first aim of this study is to provide pilot datasets on the microenvironment of larval sites. To do that, we measured several physical and biological characteristics of larval sites that have been shown to affect *Ae*. *aegypti* oviposition or larval development, including container size, shading, water pH, water salinity, temperature, humidity, the microbiome in the water, and volatile chemical compositions. Using these datasets, we then compared the microenvironment between forest larval sites (i.e., natural containers) and domestic larval sites (i.e., artificial containers). Finally, as a third aim, we examined the oviposition choices of forest and domestic Aaf toward some variables that showed significant differences between natural and artificial containers.

## 2 MATERIALS AND METHODS

### 2.1 Field study

**FIGURE 1:**
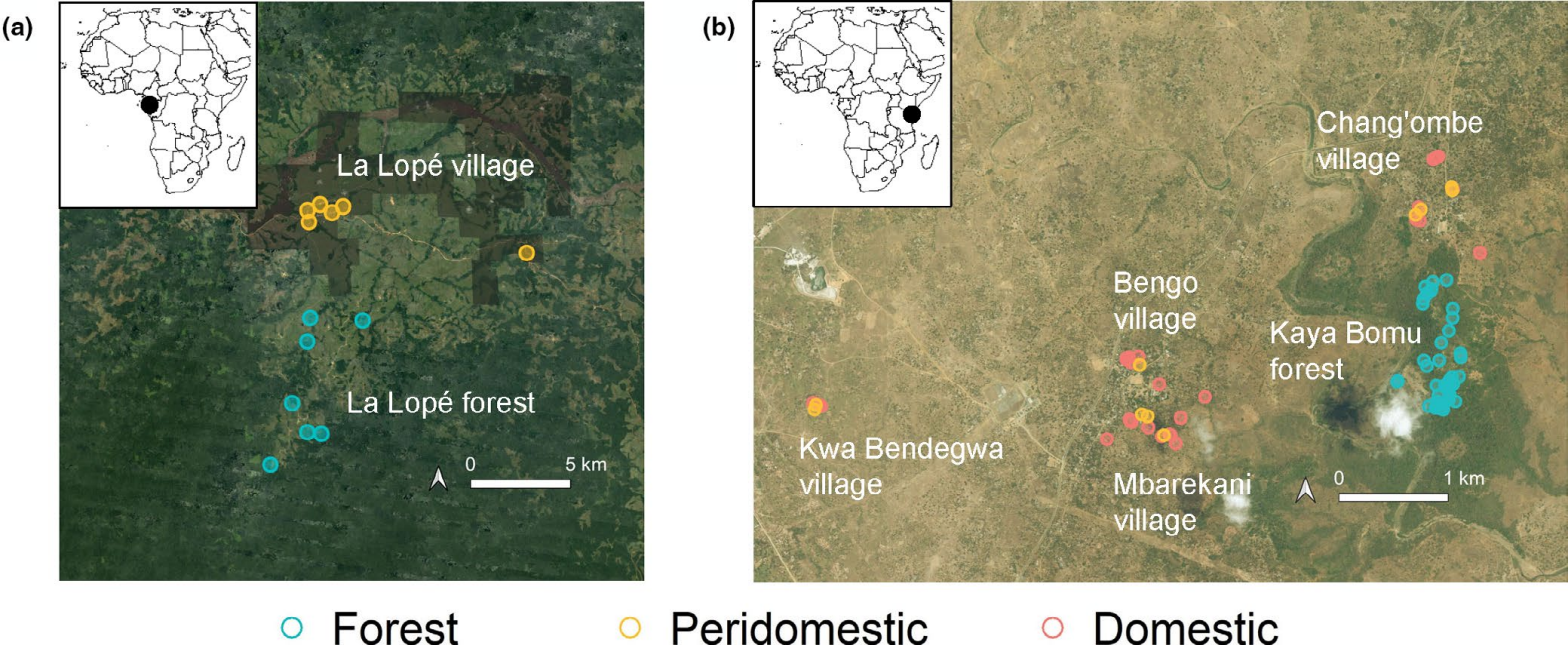
Sampling locations in (a) La Lopé, Gabon, and (b) Rabai, Kenya. The black dot in the inset in each graph shows the location of the field site in continental Africa. In (a), each point represents a sampling location where multiple larval sites were sampled. In (b), each point represents a single larval site. Colors of points indicate habitat categories: red ‐ domestic (village indoor), yellow ‐ peridomestic (village outdoor), green ‐ forest. The satellite images are from (a) Google Satellite and (b) Bing Satellite in QGIS (version 3.10.0)

We conducted field studies in La Lopé, Gabon in Central Africa from November to December 2016, and in Rabai, Kenya in East Africa from April to May 2017. The field study period overlapped with the rainy season in each locality, when *Ae. aegypti* was most abundant. La Lopé has an extensive continuous tropical rainforest surrounding the La Lopé village (Figure 1a). The forest in Rabai, on the other hand, is more fragmented, with several villages scattered around a small patch of forest (Figure 1b). In each locality, we searched for mosquito larval sites in both forests and nearby villages. A potential larval site was a container holding at least one mosquito larva (not necessarily *Ae*. *aegypti*) at the time of sampling. Overall, we sampled 60 forest larval sites and 38 village larval sites in La Lopé, and 37 forest larval sites and 31 village larval sites in Rabai (Figure 1, Table S1). Most forest larval sites in La Lopé were rock pools, while all forest larval sites in Rabai were tree holes. The village larval sites in both localities were composed of various artificial containers, including construction bricks, tires, metal cans, plastic buckets, and earthenware pots. The detailed information of all larval sites, including the GPS coordinates, can be found in the supplementary materials and online at https://doi.org/10.5061/dryad.7m0cfxprg (La Lopé) and https://doi.org/10.5061/dryad.3tx95x6cz (Rabai).

### 2.2 Characterizing physical and microbial microenvironment of larval sites

Upon identifying a larval site in any habitat, we first measured its physical characteristics. Specifically, we started by measuring the geometry of the water surface, including (1–2) two diameters, (3) the circumference, and (4) the area of the water surface. We next measured (5) the height of the container opening to the ground, (6) the container depth (only in Rabai), and (7) the water depth (only in Rabai). We also estimated (8) the water volume by collecting all water into a measuring bottle when possible. In addition to these size/volume‐related variables, we measured the ambient environment of the larval site, including (9) ambient temperature, (10) humidity, and (11) canopy coverage, using temperature and humidity loggers and a spherical densiometer. Lastly, we measured (12) water pH and (13) water conductivity in La Lopé, and three additional water characteristics in Rabai: (14) salinity, (15) total dissolved solids, and (16) water temperature. Overall, we measured 11 physical variables for each larval site in La Lopé and 16 variables in Rabai (Tables 1 and 2). The detailed methods for measuring and calculating all variables are described in Table S2.

**TABLE 1:** Summary of the 11 physical characteristics of larval sites measured in La Lopé, Gabon

	Forest *Ae*. *aegypti* present	Forest *Ae*. *aegypti* absent	Peridomestic *Ae*. *aegypti* present	Peridomestic *Ae*. *aegypti* absent
	(*n* = 5)	(*n* = 48)	(*n* = 13)	(*n* = 24)
Longest diameter (cm)	27.8 ± 17.3^a^	27.5 ± 12.5	32.7 ± 32.7	28.6 ± 27.2
Second diameter (cm)	16.1 ± 12.4	13.0 ± 6.7	22.8 ± 17.9	19.3 ± 17.8
Circumference (cm)	78.6 ± 58.7	67.9 ± 30.5	128.9 ± 158.7	97.6 ± 125.3
Surface area (cm^2^)	377 ± 410	285 ± 270	1240 ± 2323	889 ± 1820
Height of container opening (cm)	2.0 ± 4.5	8.3 ± 29.6	63.9 ± 57.2	18.6 ± 20.7
Water volume (ml)	624 ± 610	492 ± 596	16,350 ± 46,489	8677 ± 24,170
Temperature difference (°C)^b^	1.9 ± 1.9	2.7 ± 3.4	4.9 ± 2.9	4.9 ± 2.9
Humidity difference (%)^b^	−0.1 ± 6.8	−1.0 ± 9.7	−10.7 ± 6.1	−9.2 ± 8.2
Canopy coverage (%)	75 ± 24	65 ± 29	44 ± 32	57 ± 26
pH	6.38 ± 0.33	6.51 ± 0.55	6.86 ± 0.88	6.49 ± 0.44
Conductivity (µS/cm)	96.2 ± 144.3	134.9 ± 223.1	184.0 ± 213.7	103.9 ± 174.1

^a^ Mean ± standard deviation (*SD*).

^b^ Ambient temperature and humidity at the larval sites minus the temperature and humidity in the field station measured at the same time of the day.

**TABLE 2:** Summary of the 16 physical characteristics of larval sites measured in Rabai, Kenya

	Forest *Ae*. *aegypti* present	Forest *Ae*. *aegypti* absent	Peridomestic *Ae*. *aegypti* present	Peridomestic *Ae*. *aegypti* absent	Domestic *Ae*. *aegypti* present
	(*n* = 15)	(*n* = 22)	(*n* = 8)	(*n* = 1)	(*n* = 22)
Longest diameter (cm)	11.0 ± 4.5^a^	8.2 ± 3.8	36.6 ± 31.9	104	44.3 ± 12.3
Second diameter (cm)	7.7 ± 2.5	5.5 ± 2.5	28.6 ± 22.1	51	43.9 ± 13.4
Circumference (cm)	30.5 ± 11.7	21.8 ± 9.4	135.4 ± 150.8	487	139.2 ± 38.9
Surface area (cm^2^)	80 ± 52	41 ± 33	1448 ± 1996	6452	1651 ± 810
Water volume (ml)	488 ± 361	482 ± 559	12,505 ± 15,958	38,712	38,803 ± 50,210
Container depth (cm)	14.1 ± 7.9	17.7 ± 17.8	36.2 ± 31.4	28	62.0 ± 22.7
Height of container opening (cm)	67.1 ± 35.5	88.3 ± 46.6	35.2 ± 35.5	8	64.5 ± 25.0
Water depth (cm)	10.2 ± 6.3	14.7 ± 16.6	11.3 ± 9.0	6	22.5 ± 20.4
Temperature difference (°C)^b^	1.4 ± 1.8	1.3 ± 2.0	2.4 ± 2.9	9.1	1.1 ± 1.7
Humidity difference (%)^b^	0.2 ± 5.9	−0.8 ± 6.9	−2.2 ± 9.4	−14.5	0.6 ± 4.2
Canopy coverage (%)	92 ± 6	90 ± 8	75 ± 18	15	100 ± 1
pH	6.81 ± 0.75	6.55 ± 0.92	8.06 ± 0.69	7.42	8.06 ± 0.93
Conductivity (µS/cm)	1119.1 ± 849.3	712.1 ± 725.0	468.8 ± 734.4	727	618.4 ± 827.4
Salinity (ppt)	0.56 ± 0.42	0.36 ± 0.36	0.24 ± 0.36	0.36	0.31 ± 0.41
Total dissolved solids (ppm)	797.5 ± 596.0	509.3 ± 514.3	332.4 ± 520.9	516	440.0 ± 588.9
Water temperature (°C)	27.2 ± 1.0	27.8 ± 1.1	27.1 ± 2.4	27.9	26.8 ± 0.9

^a^ Mean ± standard deviation (*SD*).

^b^ Ambient temperature and humidity at the larval sites minus the temperature and humidity in the field station measured at the same time of the day.

In addition to measuring physical characteristics, we collected water samples from a subset of larval sites to analyze the microbiome in the water (Table S1). This step was performed before measuring any physical characteristics to prevent contamination. In brief, we collected 15 ml (in La Lopé) or 50 ml (in Rabai) of water samples from each larval site using sterile pipets and stored the water samples in a cooler until back to the field laboratory. To first examine the total microbial density in the water, we fixed 800 μl of each water sample with 400 μl 9% formaldehyde in the field laboratory. After returning to the lab at Yale University, we stained the fixed samples with DAPI (4′,6‐diamidino‐2‐phenylindole, Thermo Scientific, USA) to a final DAPI concentration of 5 μg/ml. We then counted the number of microbial cells using hemocytometers (DHC‐N01, INCYTO, Korea) under a widefield fluorescence microscope (Leica DMi8, Leica, German). Finally, the microbial densities were calculated by dividing the cell counts by 0.1 ml (water volume loaded onto the hemocytometers).

We used the rest of each water sample to analyze the bacterial composition via the 16s‐rRNA gene amplicon sequencing. In La Lopé, we kept the water frozen until returning to the Centre International de Recherches Médicales de Franceville (CIRMF), where we performed the DNA extraction. Bacterial cells were collected from water by centrifuge, and we extracted DNA using the QIAGEN Blood and Tissue kit. The DNA samples were then brought back to Yale University. In Rabai, we collected bacterial cells through filtration due to the lack of centrifuges in the field. Specifically, we filtered each water sample through a membrane with a pore size of 0.22 μm within 12 h of water collection. The filter membranes were kept frozen until DNA extraction in the lab at Yale University. After getting the DNA from all samples, we amplified the bacterial 16s‐rRNA gene V4 region using primers reported in Kozich et al. (2013). The amplicons from multiple samples were multiplexed with equal quantity and sequenced on Illumina MiSeq (Illumina, USA) at the Yale Center for Genome Analysis. We conducted amplicon sequencing for La Lopé and Rabai samples separately. Details on sample processing and sequencing library preparation are described in the Appendix S1.

After receiving the sequencing output, we demultiplexed the sequencing reads using USEARCH v10.0.240 (Edgar, 2010), and followed the pipeline of DADA2 (v1.8.0) (Callahan et al., 2016) to determine the bacterial community composition in each sample. DADA2 estimates sequencing errors and infers the exact sequence variants (i.e., amplicon sequence variants, or ASVs), analog to the conventional operational taxonomic unit (OTU). ASVs were blasted to the Ribosomal Database Project (RDP) 16s‐rRNA gene reference database (RDP trainset 16 and RDP species assignment 16) (Cole et al., 2014) for taxonomic assignment. The final products are two ASV composition tables, one for La Lopé and the other for Rabai, containing the read counts of each ASV in each larval site. We describe the downstream analysis in the next section.

The last components of larval site characteristics measured in this study are the volatile chemical compositions. Volatiles could act as olfactory cues for mosquitoes during oviposition (Afify & Galizia, 2015), yet volatile profiles of *Ae. aegypti* larval sites have rarely been described. As an initial attempt to fill this gap, we collected volatiles from a small number of larval sites only in Rabai, Kenya. In brief, we collected water samples from 42 larval sites (Table S1) and extracted the volatiles into an absorbent with a steady airflow for 24 h, starting each extraction within 12 h of water collection. The captured volatiles were immediately eluted with Hexane and kept frozen until returning to Yale University. We then examined the volatile samples by Gas Chromatography‐Mass Spectrometry (GC‐MS) at Yale West Campus Analytical Core, which allowed us to identify and quantify chemical compounds by mapping the GC‐MS peaks to a reference library. The technical details are described in the Appendix S1. Eventually, the GC‐MS analysis generated a dataset with concentrations of all identified chemicals from each larval site. We did not perform volatile collection in La Lopé.

Collectively, to characterize larval sites in La Lopé, we created a dataset that contains 11 physical variables, the total microbial density, and the bacterial community composition. For larval sites in Rabai, a similar dataset was generated that comprises 16 physical variables, the total microbial density, the bacterial community composition, and the preliminary volatile chemical profiles.

### 2.3 Categorization of larval sites based on habitats and *Ae. aegypti* presence

Using the datasets generated above, we proceeded to compare larval site characteristics between habitats. Larval sites were classified into three habitat groups: forest, peridomestic (outdoor containers in a village area), and domestic (indoor containers) (Table S1). We separated indoor and outdoor larval sites to be consistent with previous studies (McBride et al., 2014; Petersen, 1977), though in this study, *Ae. aegypti* indoors and outdoors were both Aaf (Xia et al., 2020). We also categorized larval sites by whether *Ae. aegypti* were present at the time of sampling (Table S1). Comparing sites with and without *Ae. aegypti* larvae could provide useful hints on *Ae. aegypti* larval habitat preference. To examine *Ae. aegypti's* presence, we collected all mosquito larvae and pupae from each larval site, reared them to adulthood, and identified the adults under a dissecting scope (Rueda, 2004). In La Lopé, all village larval sites were outdoor, that is, peridomestic, so no domestic larval sites were available. In Rabai, though we sampled all three habitats, almost all domestic and peridomestic larval sites produced *Ae. aegypti* (except one peridomestic site), so we excluded the categories “domestic‐*Ae. aegypti* absent” and “peridomestic‐*Ae. aegypti* absent” from statistical analyses.

### 2.4 Statistical analyses

In all following analyses, we compared larval site characteristics (1) across habitats regardless of *Ae. aegypti* presence status; (2) across habitats using only *Ae. aegypti* present sites; (3) between *Ae. aegypti* present and absent sites regardless of habitat; and (4) between *Ae. aegypti* present and absent sites within each habitat.

First, to analyze the physical characteristics of larval sites, we performed principal component analyses (PCA) on the 11 and 16 physical variables measured in La Lopé and Rabai, respectively. Eight sites in La Lopé were removed due to missing data. We then compared the multivariate physical environment of larval sites using multiple response permutation procedure (MRPP) with 999 permutations. In addition to physical characteristics, we compare the microbial density of larval sites in La Lopé and Rabai using Wilcoxon rank‐sum tests on log‐transformed density data.

To characterize the bacterial communities in larval sites, we first calculated their alpha diversities. Specifically, we calculated the Shannon index (Shannon, 1948) for each larval site using the ASV datasets generated by the 16s amplicon sequencing and the R package *phyloseq* (McMurdie & Holmes, 2013). We then compared the Shannon indices using the Wilcoxon rank‐sum tests. In addition, we summarized the bacterial community compositions of all larval sites by non‐metric multidimensional scaling (NMDS) with the Bray‐Curtis distance (Ramette, 2007). We removed samples with fewer than 5000 reads and thinned the reads of each sample proportionally to the lowest read depth to control for uneven sequencing depths. Bacterial communities may show different assembly patterns at different taxonomic levels (Goldford et al., 2018). Therefore, we calculated Shannon indices and performed NMDS at four taxonomic levels: ASV, species, genus, and family. Finally, we demonstrated the major bacterial families in each larval site using bar plots and used the R package *DESeq2* to identify bacterial families that were most differentiated between habitats (Love et al., 2014).

For the volatile chemical profiles of larval sites, we did not perform statistical analyses because of the sparsity of compounds in the final dataset. Instead, we summarized concentrations of all identified compounds using a heatmap.

Finally, we analyzed different types of larval site characteristics jointly by constructing random forest classification models in La Lopé and Rabai, respectively. The models aimed to predict the habitat in which a given larval site was found and whether it contained *Ae. aegypti*. The predictive variables included the scores of the first three principal components (PCs) of the physical characteristics and the first two NMDS scores from the bacterial community composition analysis. Microbial density was also included as a predictive variable in the Rabai model, but excluded in La Lopé due to missing data. The random forest models generated confusion matrices, which displayed the number of samples correctly or wrongly classified. A lower proportion of misclassification between groups suggested a stronger distinction in their environmental conditions.

All statistical analyses were performed in R v3.5.0 (R development core team, 2018). The *p*‐values for multiple comparisons were adjusted using the Holm method.

### 2.5 Temporal stability of bacterial communities in larval sites

All measurements and analyses described above were from a one‐time cross‐sectional sampling. As a preliminary attempt to examine the temporal stability of larval site microenvironments, we examined the bacterial community compositions of five larval sites in each habitat over multiple time points. To do so, we revisited these sites and collected water samples for 16s‐rRNA gene amplicon sequencing. The average interval between two consecutive collections ranges from 3 to 21 days, with an average of 8.4 days in La Lopé and 17 days in Rabai. We performed NMDS analysis to examine the bacterial composition changes between temporal samples.

### 2.6 Field oviposition experiments

During *Ae. aegypti's* transition from forest to anthropogenic habitats, a critical step was to accept novel artificial containers as larval sites. In order to examine whether *Ae. aegypti* in the forest can do so, we performed a preliminary field experiment by placing locally available artificial containers into forest areas. In La Lopé, we placed one tire, one plastic bottle, one plastic bag, one brick, and one metal can in each of four forest patches (i.e., 20 containers in total). In Rabai, we placed one plastic bucket and one earthenware pot in each of 10 forest locations (i.e., 20 containers in total). All containers were left in the forest area for 7–14 days and filled with rainwater (La Lopé) or well water from the village (Rabai). After retrieving these containers, we examined the existence of *Ae. aegypti* by rearing all larvae and pupae to adults for taxonomic identification.

### 2.7 Laboratory oviposition assays

To address our last research question on how environmental differences between natural versus artificial containers may associate with changes in oviposition preference in Aaf, we performed laboratory oviposition assays in a common‐garden setup. We first tested water samples directly collected from the field. We then focused on a few larval site characteristics that showed large differences between forest and village (peridomestic and domestic) larval sites, including pH, shading, bacterial community composition, and microbial density (see Table S3 for more details about each experiment). We also tested the combined effect of pH, salinity, and shading. Lastly, although we did not examine larval density quantitatively in the field study due to the limitation that we could only observe adults, the higher water volumes in most artificial containers (one to two magnitude higher than that of natural containers) suggested that village larval sites tended to have a much lower larval density. Therefore, we included larval density in our laboratory oviposition assays.

For all experiments, we used one *Ae. aegypti* colony established from the Rabai forest (KBO1, corresponds to the “KBO” in Rose et al. (2020)) and one from a village in Rabai (Kwa Bendegwa). Eight additional colonies from Rabai (three from the forest and five from the villages) and two colonies from La Lopé (one from the forest and the other from the village) were also tested for their oviposition preference for bacterial community composition and microbial density. All *Ae. aegypti* colonies were established from field collection. We used the fourth to the sixth generation in the oviposition assays. The detailed information of the mosquito colonies and protocols for maintaining these colonies are described in the Appendix S1 and Xia (2021).

For all oviposition assays except the one examining microbial density, we conducted two‐choice trials in which five gravid females were allowed to lay eggs in either of two oviposition cups (Figure S1a). Each cup represented either the mean forest or the mean village condition of the focal variable (Table S3). For example, when testing oviposition preference for water pH, we adjusted the pH in the two cups to match the median pH values for forest versus village larval sites in Rabai. We randomized the locations of the two cups in each cage and allowed the females to lay eggs for 24 h. Oviposition choices in each cage (i.e., a replica) were expressed by oviposition activity index (OAI) (Kramer & Mulla, 1979):

$$\text{OAI}=\frac{N_{1}‐N_{2}}{N_{1}+N_{2}},$$

where *N*_1_ and *N*_2_ are the number of eggs deposited in the two cups, respectively. OAI ranges from −1 to 1, representing a complete preference for the second choice to a complete preference for the first choice. We performed beta‐binomial models in the R package *glmmTMB* (Brooks et al., 2017) to examine whether colonies differ in their oviposition preferences, using the two egg counts in each cage as the dependent variable (Rose et al., 2020). We added the batch/trial IDs as random effects if data testing a specific condition were generated from more than one experimental batch. The statistical significance of colony or habitat effects was determined by comparing the full model with a null model that excluded colony or habitat (Table S10). We extracted mean OAI with a 95% confidence interval from the model using the R package *emmeans* (Lenth et al., 2018; Rose et al., 2020).

In the experiment on oviposition preference for microbial density, we used five bacterial densities in each cage instead of the two‐choice design (Figure S1b). This new design allowed us to examine a broader range of bacterial density observed in larval sites (more than two orders of magnitude). The five bacterial densities ranged from zero to 2.5 × 10^7^ cells/ml, roughly the maximal bacterial density observed in the field study (Table S3). After counting the numbers of eggs laid in the five cups, we fitted a negative‐binomial model using the R package *lme4* (Bates et al., 2015), with bacterial densities, habitats or colonies, and their interactions as predictors. If *Ae. aegypti* from different colonies or habitat types have different oviposition choices, the interaction term would be significant, which was tested by comparing the full model with a null model excluding the interactive term (Table S10). We added cage ID as a random effect. Lastly, we used the *emmeans* package to estimate the expected number of eggs in each bacterial density with a 95% confidence interval.

## 3 RESULTS

### 3.1 Dataset of the physical and microbial microenvironment of larval sites

Datasets of larval site physical characteristics and microbial density in La Lopé and Rabai can be found in Supplementary materials S1 and S2, respectively. They were also deposited in the public database: https://doi.org/10.5061/dryad.7m0cfxprg (La Lopé physical characteristics) and https://doi.org/10.5061/dryad.3tx95x6cz (Rabai physical characteristics and volatile chemical profiles). The mean and standard deviations of each variable are summarized in Table 1 (La Lopé) and Table 2 (Rabai). The 16s sequencing reads are deposited in the NCBI SRA database with ID PRJNA644189 (La Lopé) and PRJNA644205 (Rabai).

### 3.2 Comparison of larval site microenvironment

**FIGURE 2:**
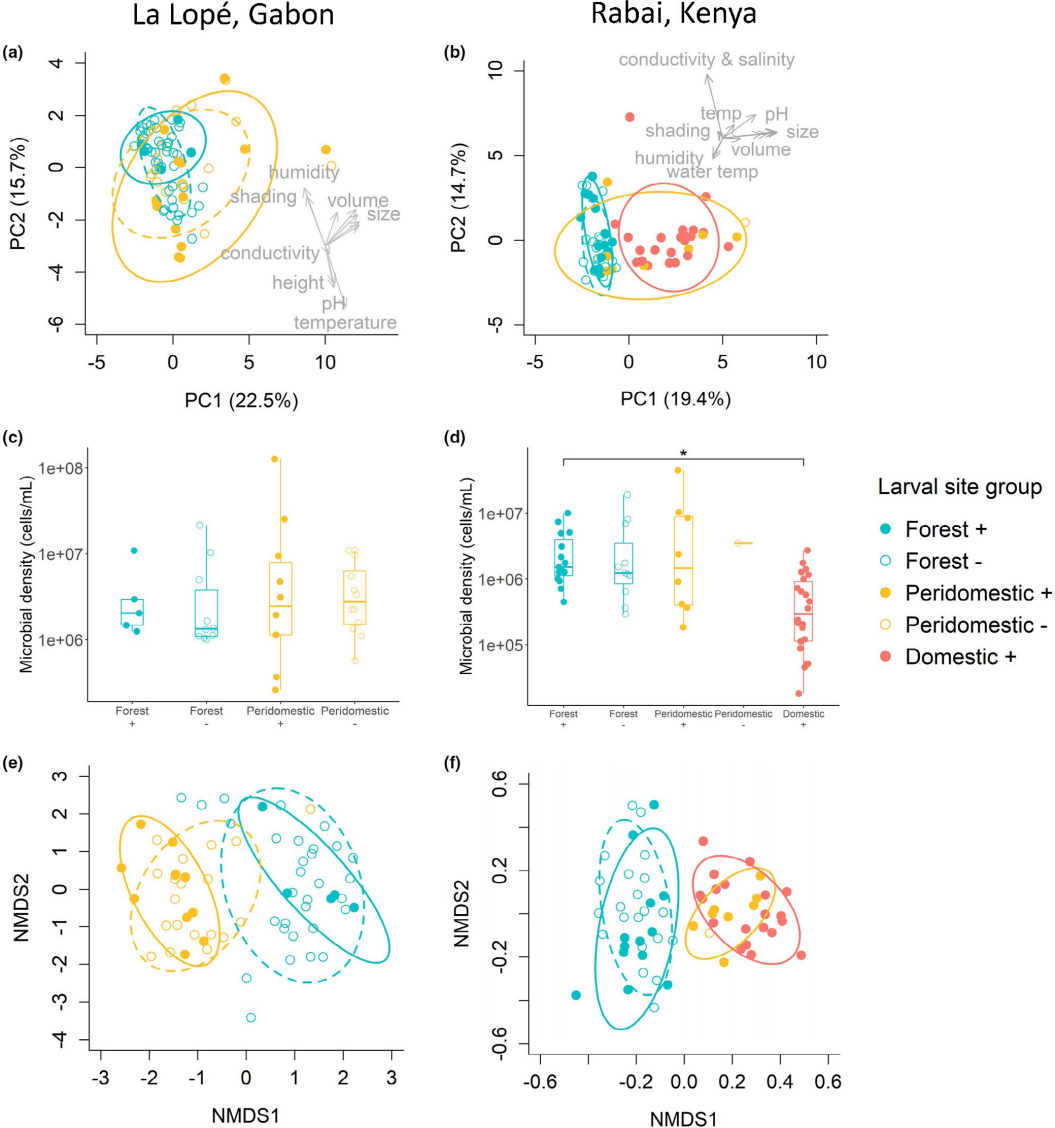
Physical and microbial characteristics of larval sites in La Lopé (a, c, e) and Rabai (b, d, f). (a‐b) Principal component analysis (PCA) of all physical variables of larval sites. The first two PCs are shown, and the variance explained by each PC is indicated in the axis label. Each point represents a single larval site. Colors of points indicate habitat, and shapes of points indicate whether *Ae. aegypti* were found in the sites (i.e., *Ae*. *aegypti* presence status). In the legend, “+” and “−” denote *Ae. aegypti* present and absent sites, respectively. An ellipse is drawn for each larval site group with a 75% confidence level. The colors of ellipses represent habitat types and match the colors of points. The solid and dashed ellipses correspond to *Ae. aegypti* present and absent sites. The original variables are overlaid as vectors on the PC1‐PC2 plate with major variables labeled. The vectors indicate the linear coefficients of the original variables (i.e., the loadings) on the first two PCs. (c‐d) Comparison of microbial densities of larval sites. The boxplots show the minimum, 25% quartile, median, 75% quartile, and maximum. Asterisks indicate statistically significant differences (*p *< .05). (e‐f) Non‐metric multidimensional scaling (NMDS) analysis of bacterial community compositions. The colors and shapes of points and ellipses are the same as in the PCA (panel a‐b)

We used PCA to characterize the physical characteristics of larval sites. In La Lopé, larval sites in different habitats overlap extensively in the space described by the first two principal components, which together account for 38% of the total variance (Figure 2a). However, MRPP tests showed significant multivariate differences between forest and peridomestic habitats (Table 3). Examination of the loading of the original variables on each of the two PC axes shows that forest sites tended to be cooler, more humid, better shaded, and closer to the ground (see also Table 1). We observed no difference between larval sites with and without *Ae. aegypti*, either within each habitat or across habitats (Figure 2a, Table 3).

**TABLE 3:** Comparison of larval site physical characteristics

Comparison^a^	La Lopé			Rabai		
	*δ* ^b^	*E*(*δ*)^b^	*p* ^b^	*δ* ^b^	*E*(*δ*)^b^	*p* ^b^
Forest vs. peridomestic	8873	9375	.**005**	4986	6566	.**008**
Forest vs. domestic	n.a.^c^	n.a.	n.a.	18,970	25,424	.**008**
Peridomestic vs. domestic	n.a.	n.a.	n.a.	40,384	41,099	.441
Forest + vs. peridomestic +	22,056	22,509	.280	7180	8626	.**012**
Forest + vs. domestic +	n.a.	n.a.	n.a.	29,491	36,206	.**008**
Peridomestic + vs. domestic +	n.a.	n.a.	n.a.	40,523	41,604	.441
*Ae. aegypti* + vs. *Ae*. *aegypti* −	9356	9375	.918	23,248	24,833	.**008**
Forest + vs. forest −	759	752	1	1286	1306	0.441
Peridomestic + vs. peridomestic −	20,864	20,505	1	n.a.	n.a.	n.a.

^a^ “+” and “−” denote *Ae. aegypti* present and absent sites, respectively.

^b^ Results of multiple response permutation procedure (MRPP) with 999 permutations. *δ*: overall weighted mean of within‐group means of the pairwise dissimilarities; *E*(*δ*): expected *δ* under the null hypothesis of no group structure; *p*: *p*‐values after Holm adjustment of multiple comparisons, with boldness indicating statistical significance (*p* < .05).

^c^ Comparisons were not available as no domestic larval sites were sampled in La Lopé, and all but one peridomestic and domestic larval sites in Rabai were *Ae. aegypti* present (+).

In Rabai, forest and domestic larval sites showed a clear distinction in their physical characteristics, with peridomestic larval sites overlapping with both forest and domestic sites (Figure 2b). The differences between habitats were primarily driven by domestic sites having larger sizes, higher water volume, and higher water pH (i.e., more alkaline) (Table 2). MRPP found significant multivariate differences between forest and the other two habitats but no difference between peridomestic and domestic larval sites (Table 3). Larval sites with and without *Ae. aegypti* again showed little differences (Figure 2b, Table 3).

Microbial density was not significantly different between habitats or between sites with and without *Ae. aegypti* in La Lopé (Figure 2c, Table S4). In Rabai, domestic larval sites showed a significantly lower microbial density than forest sites (Figure 2d, Table S5). In both localities, *Ae. aegypti* present and absent sites had comparable microbial densities (Tables S4 and S5).

The alpha diversity of bacterial communities in larval sites was generally comparable across habitats and between sites with and without *Ae. aegypti* in both La Lopé and Rabai (Tables S4 and S5, Figures S2 and S3). The only significant difference was that in La Lopé, peridomestic larval sites had a higher Shannon index than forest sites at the species and genus level (Table S4).

We next summarized the bacterial community compositions by NMDS. At the ASV level, forest larval sites had a very different bacterial community from peridomestic and domestic larval sites, both in La Lopé (Figure 2e) and Rabai (Figure 2f). This striking divergence between habitats was less evident at higher taxonomic levels in La Lopé (Figure S4), while the Rabai samples retained the difference at all four taxonomic levels (Figure S5). The bacterial compositions in peridomestic versus domestic larval sites in Rabai were almost indistinguishable (Figure 2f). Within each habitat, larval sites with and without *Ae. aegypti* shared similar bacterial community composition in both localities (Figure 2e,f). The most abundant bacterial families varied considerably among samples (Figure S6). Most larval sites contained multiple bacterial families with no clear dominance. However, *DESeq2* found some bacterial families showing differential abundance between habitats (Tables S6 and S7).

**FIGURE 3:**
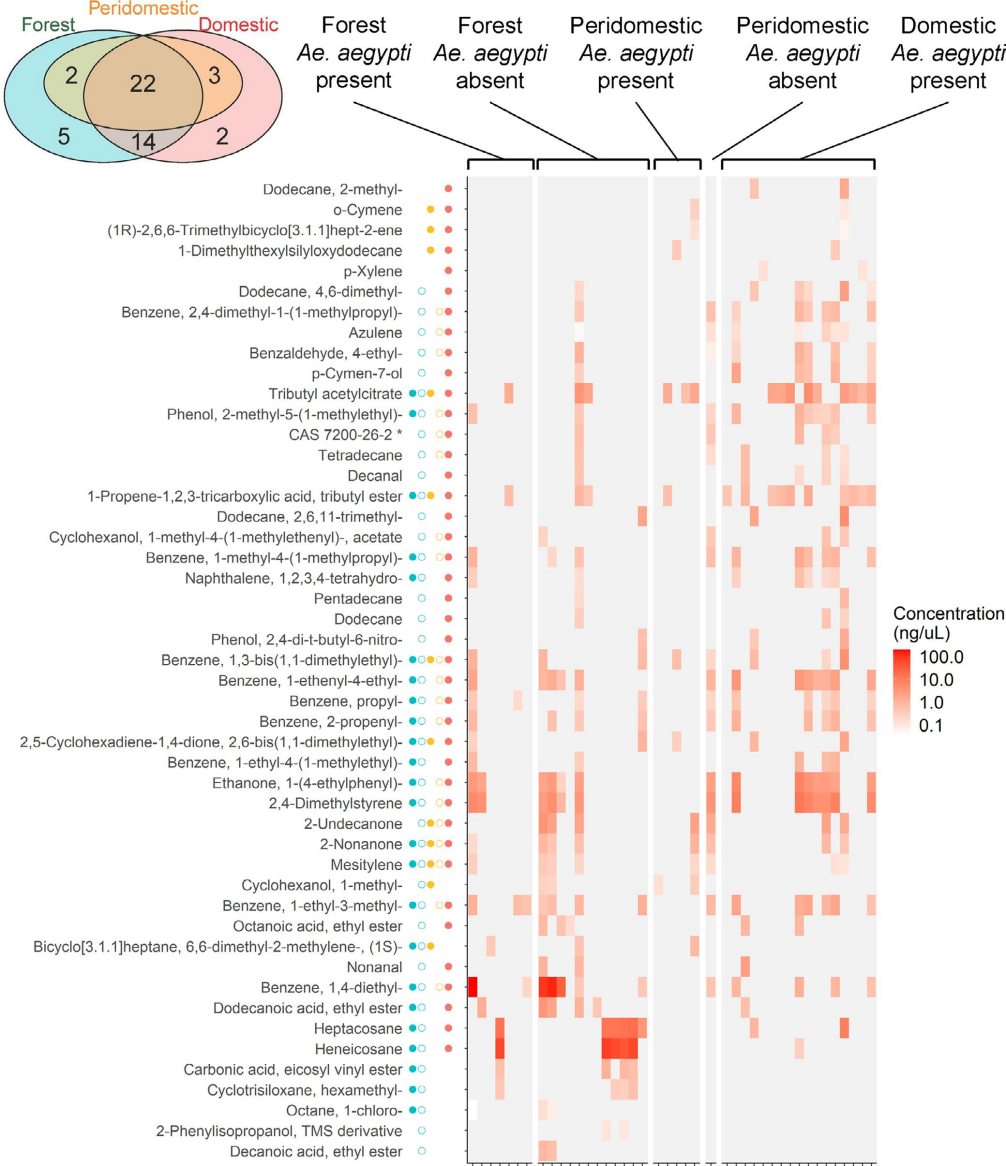
Volatile chemical profiles of Rabai larval sites. Each row represents a chemical compound, and each column represents a larval site. The five columns of points between the compound names and the heatmap summarize whether the compounds were present in each of the five larval site groups. The colors and shapes of points are the same as in Figure 2. The color of each cell in the heatmap quantifies the concentration on a log scale. Gray cells indicate that the compounds were not found in the larval sites. The inset Venn diagram shows the total numbers of chemical compounds unique to each habitat and shared between different habitats

The volatile profiles of 31 larval sites in Rabai are demonstrated in Figure 3. There were substantial variations in the chemical composition of samples, both within habitats and across habitats. GC‐MS analysis identified a total of 29 chemical compounds. Most of them were shared across habitats, but a few chemicals were unique to either forest or domestic habitat (Figure 3, top five rows and bottom five rows).

Lastly, the random forest models constructed with both physical and microbial characteristics showed high accuracy in distinguishing larval sites in forests versus villages (peridomestic and domestic) in both La Lopé and Rabai (Table 4, see also Tables S8 and S9 for the confusion matrices of La Lopé and Rabai data, respectively). Rabai peridomestic and domestic samples were less accurately classified, consistent with the finding that they shared similar physical characteristics and bacterial community compositions. Within each habitat, the classification algorithm distinguished sites with and without *Ae. aegypti* poorly (Tables S8 and S9).

**TABLE 4:** Misclassification of larval site habitats and *Ae. aegypti* presence statuses by random forest models using physical and microbial characteristics

Locality	Dependent variable	Total misclassification	Habitat misclassification	*Ae*. *aegypti* presence status misclassification
La Lopé (*n* = 63)	Habitat	6% (4/63)	6% (4/63)	n.a.^c^
	*Ae*. *aegypti* presence	25% (16/63)	n.a.	25% (16/63)
	Larval site group^a^	32% (20/63)	6% (4/63)	25% (16/63)
Rabai (*n* = 52)	Habitat	12% (6/52)	12% (6/52)	n.a.
	Forest vs. village^b^	0% (0/52)	0% (0/52)	n.a.
	*Ae*. *aegypti* presence	31% (16/52)	n.a.	31% (16/52)
	Larval site group^a^	35% (18/52)	10% (5/52)	25% (13/52)

^a^ Combinations of habitats and *Ae. aegypti* presence statuses (e.g., “forest‐*Ae. aegypti* present”).

^b^ Village larval sites contain both peridomestic and domestic larval sites.

^c^ Not applicable.

### 3.3 Temporal stability of bacterial communities in larval sites

NMDS analysis found that temporal samples collected from the same larval breeding sites did vary in their bacterial community compositions. However, these temporal changes did not exceed the variations observed within each habitat (Figure S7). Temporal samples from the forest still clustered with the rest of the forest sites instead of samples from the village, and vice versa.

### 3.4 Field oviposition experiments

In La Lopé, the 20 artificial containers we placed in the forest produced in total 22 *Ae. aegypti*, with 15 *Ae. aegypti* from tires, four from plastic bottles, two from metal cans, and one from plastic bags. In Rabai forest, we found 546 *Ae. aegypti* from eight plastic buckets and 99 *Ae. aegypti* from six earthenware pots.

### 3.5 Laboratory oviposition assays

**FIGURE 4:**
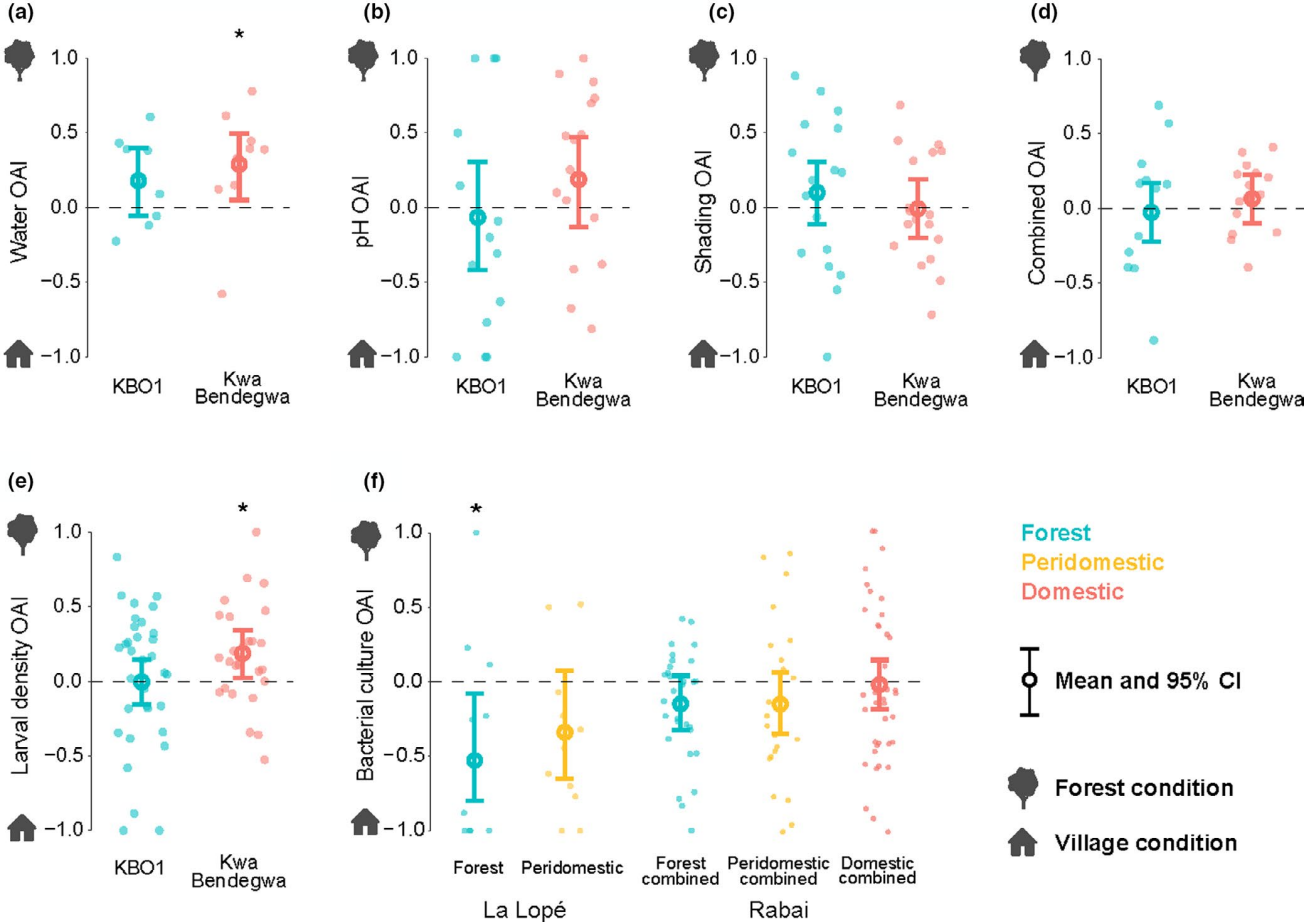
Two‐choice laboratory oviposition assays testing oviposition preference for (a) field‐collected waters, (b) water pH, (c) shading, (d) a combination of water pH, salinity, and shading, (e) *Ae. aegypti* larval density, and (f) bacterial culture. Colony‐wise results are shown in Figure S8. The two choices in each assay represent the forest and village larval site characteristics (described in Table S3). Higher OAI implies a preference for the forest condition. Each point represents the OAI of one cage with five gravid females. The mean and 95% confidence interval (CI) were estimated by beta‐binomial models. The asterisk indicates that the 95% CI excludes zero. No significant differences are found between colonies or habitats of the colonies in any experiment

**FIGURE 5:**
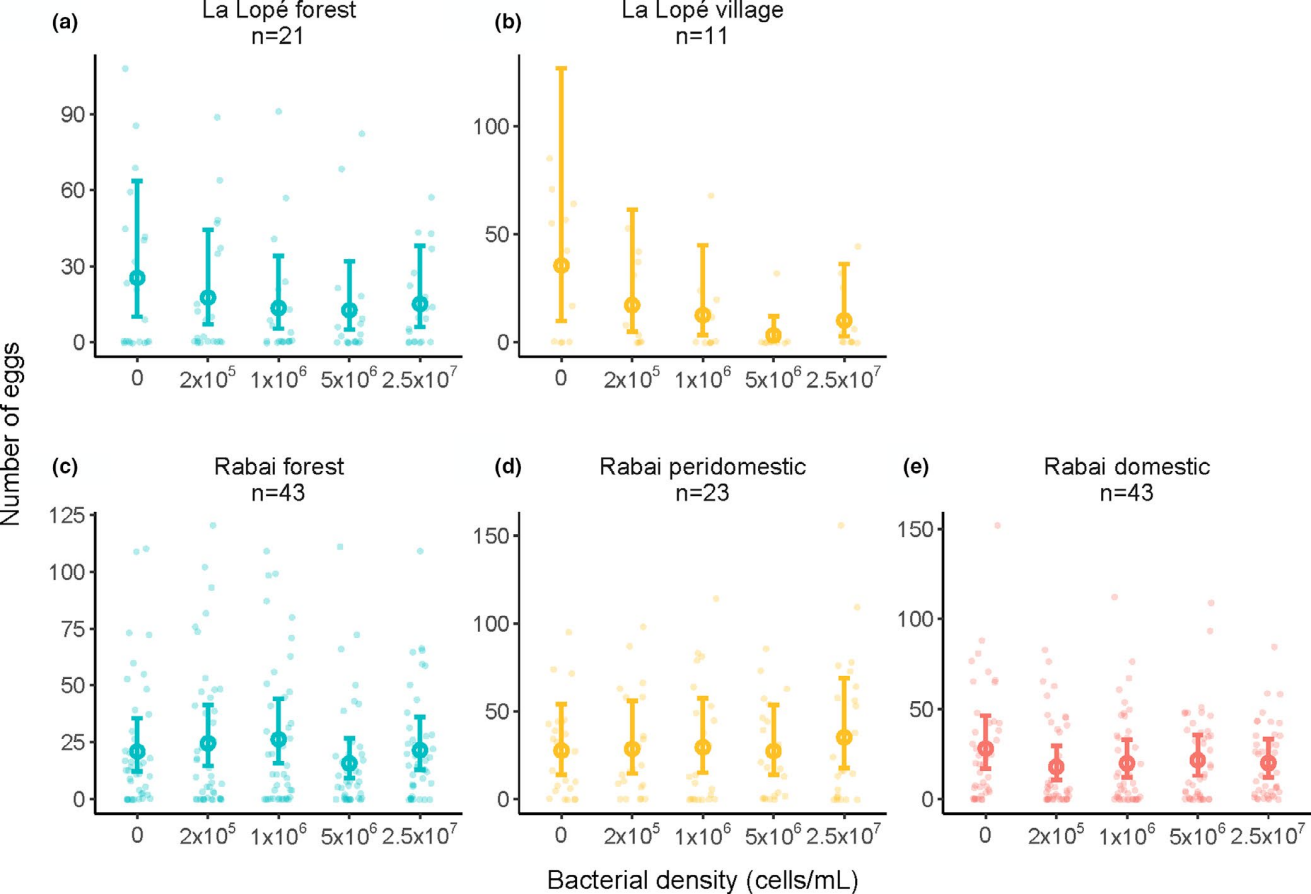
Five‐choice laboratory oviposition assays testing oviposition preference for bacterial density. Each data point represents the number of eggs laid in a specific bacterial density in one cage. Colors represent the habitats of the colonies. Multiple colonies from the same habitat are combined in this figure, and colony‐wise results are shown in Figure S9. A negative binomial model was used to estimate the mean number of eggs in each bacterial density and a 95% confidence interval, shown by the open circles and error bars

Among all two‐choice oviposition experiments, we found three significant preferences (Figure 4 and Figure S8): (1) Rabai Kwa Bendegwa village colony preferred forest water over village water; (2) The same village colony also preferred higher conspecific larval density (characteristic of forest larval sites in the field); (3) La Lopé forest colony preferred the bacterial culture started with peridomestic water samples. There were substantial within‐colony variations in most experiments. The beta‐binomial models did not find any significant difference in oviposition preference between colonies or habitats in any assay (Table S10). In the five‐choice oviposition assays testing preferences for bacterial densities, the negative‐binomial model showed that neither mosquito colonies nor the habitats of the colonies had a significant effect on the mosquito's choice (Figure 5 and Figure S9, Table S10).

## 4 DISCUSSION

### 4.1 Larval site microenvironment and its implication on Aaf habitat change

In this study, we first acquired data describing the physical and microbial microenvironment of larval sites in La Lopé, Gabon, and Rabai, Kenya. These datasets add to previous studies that characterize larval habitats of African *Ae*. *aegypti*, such as Dickson et al. (2017) and Onchuru et al. (2016), which collectively provide fundamental ecological information for studying Aaf larval ecology and evolution.

Using our datasets, we demonstrated that Aaf larval sites in the forest and villages (including peridomestic and domestic sites) had different physical and microbial characteristics. However, factors accounting for between‐habitat contrasts varied in the two localities. For instance, in La Lopé, larval sites differed strongly on their ambient environments, such as temperature, humidity, and shading. The forest sites were generally cooler, more humid, and better shaded. They were also closer to the ground, as most forest larval sites were rock pools. On the other hand, in Rabai, temperature and humidity were comparable across habitats. The most striking difference was that village larval sites tended to be much larger. They also had much lower bacterial density. These results were expected, as most larval sites in the Rabai villages were artificial containers used by villagers to store water. We also did not find many other mosquito species in these artificial containers, though we did not quantify species composition. This potential lack of interspecific competition, combined with the low microbial density that suggested low larval food availability, may present different selection pressures at the larval stage for village‐living Aaf. For example, it may affect the evolution of larval starvation resistance (Barrera & Medialdea, 1996; Souza et al., 2019). Interestingly, we also found a higher water pH in peridomestic and domestic larval sites in Rabai compared to forest sites. How that might affect Aaf larvae is unknown.

Despite these differences in larval site characteristics, behavioral investigations suggested that Aaf in the forest readily accepted artificial containers as oviposition and larval sites. In laboratory studies, *Ae. aegypti* colonies derived from the forest and villages showed little consistent difference in oviposition preferences when presented with choices mimicking the larval characteristics in forest versus village larval sites. Oviposition responses were also highly heterogeneous among individual cages. These results are consistent with the hypothesis that Aaf is a generalist in oviposition choice. This hypothesis is also supported by the indistinguishable conditions between *Ae. aegypti* present and absent larval sites within each habitat. Being versatile in larval habitats may allow forest *Ae*. *aegypti* to take advantage of novel artificial containers when natural breeding sites are scarce, which has been proposed as a key driver for this species to move into domestic habitats in the first place (Brown et al., 2014; Powell et al., 2018; Rose et al., 2020). Indeed, *Ae. aegypti* in La Lopé and Rabai likely could easily move between forests and villages, as suggested by studies showing little genetic differentiation between forest and village *Ae*. *aegypti* populations (Kotsakiozi et al., 2018; Paupy et al., 2014; Rose et al., 2020; Xia et al., 2020). However, because this study did not examine fitness or larval productivity directly, we cannot rule out the possibility that larval survival differed in forest and village larval sites, as oviposition preference does not always align with larval survival and development (Refsnider & Janzen, 2010). Aaf in different habitats may also have evolved incipient divergences in oviposition or larval adaptations not detected in this study.

### 4.2 Limitations

While our larval site characteristic datasets could provide useful information for future studies on *Ae. aegypti* ecology and behavior, we acknowledge that some caveats exist in our field sampling and measurements. Therefore, the results should be interpreted with caution. Most importantly, we only sampled most larval sites once during a narrow temporal window (several weeks) in the rainy season. This snapshot sampling may not capture the full dynamics of the larval site microenvironments, e.g., the seasonal variations. Although we showed that the temporal variations of the microbial community in larval sites over several weeks did not exceed the between‐habitat differences, they could still change drastically over a longer period. Future studies are needed to monitor larval site microenvironments throughout the year, as recent work suggested the importance of seasonality in driving the domestication of *Ae. aegypti* (Rose et al., 2020). Second, we grouped tree holes and rock pools as “natural” containers due to the limitation of sample sizes, yet previous studies have implied that they could be two distinct larval habitats (Soghigian et al., 2017). However, our preliminary analysis suggested that separating them did not affect the main findings from the field study. We also acknowledge that the chemical profiles of larval sites in Rabai reported in this study were probably not complete or very accurate, due to our limited equipment and chemical collection experience in the field. We hope future studies with improved sample collection and analysis techniques could provide a more comprehensive and precise profile of larval site chemical compositions. Lastly, the local context of a larval site could also be important, for example, vegetation around the site (Rey & O'Connell, 2014), but was not characterized in this study.

In our comparisons of larval site characteristics across habitats and sites with and without *Ae. aegypti*, it is important to point out that the absence of *Ae. aegypti* in a larval site did not necessarily reflect avoidance by the female nor that it is inhospitable for the larvae, but simply due to chance. In addition, we could not count *Ae. aegypti* eggs, larvae, or pupae directly, which prevented us from measuring larval density, survival, or productivity to draw any conclusion on fitness. We also had a relatively small sample size in our field study, at least for some larval site groups. For instance, we found only five larval sites in La Lopé forest with *Ae. aegypti*. These small sample sizes might hinder our ability to detect differences in larval site characteristics across habitats or between sites with and without *Ae. aegypti*.

Finally, while our laboratory oviposition experiments did not find strong oviposition preferences, we cannot rule out the possibility that our experimental design lacked the power to detect subtle oviposition preference or differences between colonies. The two‐choice or multi‐choice assays have been used widely to investigate *Ae. aegypti* oviposition choices, but it may have limitations on measuring true preferences (Singer, 2004, 2021). Colonies may have also lost distinctive traits due to adaptation to laboratory conditions (Hoffmann & Ross, 2018). Moreover, the design of using five females per cage instead of one female might introduce some unknown complexity, for instance, interferences between individuals (Allan & Kline, 1998). Lastly, the contrast of oviposition choices might not be of a magnitude detectable by female *Ae. aegypti*. However, the choices used in this study were informed from the field study, and therefore should be ecologically relevant for the mosquitoes. A recent study using the same *Ae. aegypti* colonies did find ovipositional differences between forest and village colonies toward more extreme but unnatural conditions (Xia, 2021), highlighting the complexities of *Ae. aegypti* oviposition.

## 5 CONCLUSIONS AND IMPLICATIONS

In summary, this study characterized Aaf larval site microenvironments and suggested that *Ae. aegypti* in Africa were likely generalists in larval habitats, and by implication, generalists in oviposition preferences. This might allow them to readily accept artificial containers as larval sites and potentially facilitate their introduction into domestic habitats. Interestingly, this hypothesis was echoed by a recent study on another mosquito species, *Anopheles coluzzii*, a primary vector of malaria in Africa. The study found that *An*. *coluzzii* can colonize larval habitats with a wide range of physicochemical conditions, likely associated with the species' dominance in urban settings in Africa (Longo‐Pendy et al., 2021).

Outside of Africa, *Ae. aegypti* are closely associated with human communities and use almost exclusively artificial containers for larval sites (Day, 2016; Swan et al., 2018; Vezzani, 2007; Yee, 2008), raising the interesting question of when and how this reliance on artificial containers evolved. A few recent studies suggested that human adaptations may have happened somewhere in West Africa, such as Sahel or Angola (Crawford et al., 2017; Powell et al., 2018; Rose et al., 2020). On the other hand, Aaa outside Africa evidently can revert to ancestral larval habitats, for instance, in the Caribbean (Chadee et al., 1998). It would be interesting to examine such processes and test whether these Aaa populations reverted to being more generalists in larval sites compared to places where Aaa is solely using artificial containers.

## CONFLICT OF INTEREST

The authors declare that they have no conflict of interest.

## AUTHOR CONTRIBUTIONS

**Siyang Xia:** Conceptualization (lead); data curation (lead); formal analysis (lead); investigation (lead); methodology (lead); project administration (lead); resources (equal); validation (lead); visualization (lead); writing‐original draft (lead); writing‐review & editing (lead). **Hany K. M. Dweck:** Data curation (supporting); formal analysis (equal); methodology (equal); software (equal); validation (supporting); writing‐review & editing (supporting). **Joel Lutomiah:** Conceptualization (supporting); investigation (supporting); methodology (supporting); project administration (equal); resources (equal); supervision (supporting). **Rosemary Sang:** Investigation (supporting); project administration (supporting); resources (equal). **Carolyn S. McBride:** Conceptualization (equal); investigation (equal); methodology (supporting); project administration (equal); supervision (equal); validation (supporting); visualization (supporting); writing‐review & editing (equal). **Noah H. Rose:** Investigation (supporting); methodology (supporting); project administration (supporting); software (supporting); validation (supporting); writing‐review & editing (supporting). **Diego Ayala:** Conceptualization (equal); investigation (supporting); methodology (equal); project administration (equal); resources (equal); supervision (equal); writing‐review & editing (supporting). **Jeffrey R. Powell:** Conceptualization (equal); funding acquisition (lead); investigation (equal); project administration (equal); resources (lead); supervision (lead); validation (equal); writing‐review & editing (equal).

### OPEN RESEARCH BADGES

This article has earned an Open Data, for making publicly available the digitally‐shareable data necessary to reproduce the reported results. The data is available at https://doi.org/10.5061/dryad.7m0cfxprg and https://doi.org/10.5061/dryad.3tx95x6cz.

## Supporting information

Supplement S1

Supplement S2

Appendix S1

## Data Availability

The datasets that describe the basic information, physical characteristics, microbial densities, and chemical profiles of oviposition sites in La Lopé and Rabai are archived in Dryad: https://doi.org/10.5061/dryad.7m0cfxprg (La Lopé) and https://doi.org/10.5061/dryad.3tx95x6cz (Rabai), respectively. The 16s‐rRNA gene amplicon sequencing data are deposited in the NCBI SRA database with ID PRJNA644189 (La Lopé samples) and PRJNA644205 (Rabai samples).
