# Supplementary material for: Larval sites of the mosquito Aedes aegypti formosus in forest and domestic habitats in Africa and the potential association with oviposition evolution
Source: Ecol Evol. 2021 Nov 9;11(22):16327–43. doi: 10.1002/ece3.8332 (PMC8601902; doi:10.1002/ece3.8332)
Supplement: Supplementary file 3 — Appendix S1 [file ECE3-11-16327-s002.docx]

Appendix

**Method details**

*16s-rRNA gene amplicon sequencing of bacterial samples collected from larval sites*

In La Lopé, after aliquoting a subset of water samples into formaldehyde solution for microbial density measuring, we kept the remaining water at -20 °C in the field until returning to Centre International de Rrecherches médicales de Franceville (CIRMF), Franceville, Gabon. Then, we centrifuged the samples after thawing using a Backman TJ-6 centrifuge (Beckman Coulter, USA) at 6000 rpm (maximum speed) for 30 minutes to collect microbial cells. The liquid was removed immediately after centrifuge, and we extracted DNA using the QIAGEN Blood and Tissue kit (QIAGEN, USA) following the manufactural protocol. The DNA was stored at -20 °C until brought back to the lab at Yale University. In Rabai, due to lack of access to centrifuge, we instead used a filtering approach to collect microbiome from water samples. Specifically, upon bringing the water samples back to the field station, we pushed around 50 mL water from each sample using a sterile syringe through a Millipore Sterivex filter unit (SVGPL10RC, EMD Millipore, USA) with 0.22 µm pore size to collect microbial cells. We then sealed the filtering unit and frozen them at -20 °C until bringing them back to Yale University. In the lab, we then pealed the members from the filter units under aseptic conditions and extracted DNA from them using the DNeasy PowerWater Kit (QIAGEN, USA) according to the manufactural protocol.

To prepare sequencing libraries, we followed the Kozich et al. (2013) protocol. The protocol amplified the V4 region of the bacterial 16s-rRNA gene with duel indexes, which allows us to multiplex multiple samples in one sequencing. We generated two libraries, one for La Lopé bacterial samples and one for Rabai samples. The PCR primers were prepared in Dr. Andrew Goodman’s lab at Yale University as Gülden et al. (2017). We amplified the bacterial using the Q5 High-Fidelity DNA Polymerase (New England Biolabs Inc., USA) with a similar PCR cycle in Kozich et al. (2013). The amplification products were cleaned using SPRI beads (AMPure XP, Beckman Coulter, USA). We then determined the DNA concentration of each sample by Qubit (Qubit Fluorometric and dsDNA HS Assay Kit, Thermal Fisher, USA) and mixed the samples with equal quantities. To evaluate the sequencing and analysis performance, we added four positive controls into the mixed sequencing library, including two genomic DNA from Microbial Mock Community B (HM-276D and HM-277D, BEI Resources, NIAID, NIH as part of the Human Microbiome Project) (Nelson et al., 2014), one genomic DNA from ZymoBIOMICS (D6305), and one mixed community cells from ZymoBIOMICS (D6300). The final library was examined on Bioanalyzer (Thermo Fisher, USA) to confirm the amplicon size and sent to the Yale Center for Genome Analysis for Illumina MiSeq (Illumina, USA) 250 bp pair-ended sequencing.

The sequencing results were demultiplexed and analyzed as described in the main document. The median depth of the amplicon sequencing was 17,420 reads per La Lopé sample and 56,478 reads per Rabai sample. Negative controls yielded 0 - 976 reads (median: 33) and 0 - 11 ASVs (median: 4) per sample, which suggested minimal contamination from the sampling and library preparation procedures. We also reconstructed the mock communities relatively well using the sequencing results: we identified 18-23 ASVs from HM-276D and HM-277D, which contain 20 bacterial taxa. For the ZymoBIOMICS mock communities that contain eight bacterial colonies, we identified nine ASVs.

*Characterizing the volatile chemical profiles of larval sites in Rabai, Kenya*

We collected 8-15 mL water from a subset of larval sites (Table S1) into glass vials that were previously washed and baked at 400 °C. An air pump (Casella Apex Pro, UK) was used to generate an airflow at 0.2 L/min to extract the volatiles from the water in the vials for 24 hours. The chemicals in the airflow leaving the vials were captured using Volatile Collection Trap (Volatile Collection Trap LLC, USA) with PoraPak-Q and later eluted in 200 μL Hexane (Sigma-Aldrich, USA). 1-Bromoheptane (B67570, Sigma-Aldrich, USA) was mixed in the Hexane as an internal standard with a concentration of 100 ng/μL. We started volatile extraction on the evening of each collection day, less than 12 hours since the water collection. An empty vial was used every day as a negative control to characterize the background chemical profile in the airflow. After elusion, the Hexane solutions were kept at -20 °C until shipped back to Yale University.

To analyze the chemical profiles in the volatile samples, we injected 1 μL of each sample into the gas chromatography-mass spectrometry system (GC-MS, Agilent 7890A/5975C, Agilent Technologies, Inc. USA) at Yale West Campus Analytical Core. We analyzed the GC-MS results using MSD ChemStation F.01.03.2357 (Agilent Technologies, Inc. USA). We first identified any compounds that exist only in the samples or have a substantially higher quantity in the samples compared to the corresponding negative control (volatiles collected from the empty vial in the field). The chemical compounds were then identified using the National Institute of Standards and Technology (NIST) reference library (v 2.2, Scientific Instrument Services, USA). We quantified each compound’s concentrations using the area underneath the corresponding peak(s) and used the 1-Bromoheptane peak to translate area to absolute concentration. We removed compounds found in only one larval site, which could result from identification errors or contamination. We also excluded compounds that do not exist in nature (http://www.thegoodscentscompany.com), which suggested contamination or misidentification. In total, 29 chemical compounds were identified. We then removed 11 larval sites that did not have any of the 29 compounds.

*Mosquito colonies*

*Aedes aegypti* collected from natural larval sites were kept alive in the field to establish colonies. In La Lopé, due to the low number of *Ae. aegypti* collected, we pooled all individuals from each habitat to establish a single forest colony and a single village colony. We also distributed several bamboo traps and performed human landing catches in La Lopé forest to supplement the larval collection. In Rabai, all colony-forming *Ae. aegypti* came from natural breeding sites. We established two forest colonies (one from the deep area in the forest and another from the edge of the forest adjacent to the Chang’ombe village) and four domestic colonies collected from domestic larval sites in the four villages (Kwa Bendegwa, Bengo, Mbarekani, and Chang’ombe). In both La Lopé and Rabai, we blood-fed the females in the field multiple times and collected eggs (the second generation) on seed germination papers (SD7606, Anchor Paper Company, USA). After producing eggs, *Ae. aegypti* were preserved in ethanol for genetic analyses (reported in Xia et al. 2020).

The eggs of the two La Lopé colonies and the four Rabai domestic colonies were brought back to Yale University at the end of the fieldwork (USDA Veterinary Permit 103356). The two Rabai forest colonies were first brought back to the McBride Lab at Princeton University and maintained as described in Rose et al. (2020). A copy of these two colonies (KBO1 and KBO2 in Rose et al. (2020), also coded as K66 and K67) in the third generation was sent back to our lab at Yale University, while the original copy was continued at the McBride Lab. We kept the mosquitoes in the insectary with a 27 ˚C constant temperature, a 50%-70% relative humidity, and a 12h/12h light/dark cycle. Eggs were hatched with deionized water supplied with fish food (TetraMarine Saltwater Granules, Tetra, German), and pupae were transferred into insect rearing cages (BugDorm-1) that are roughly 30 x 30 x30 cm. We provided 10% sugar water to adults constantly and fed them multiple times with sheep blood (DSB050, Hemostat, USA) at least five days after they emerged. Three days after feeding, we provided four cups (two black cups and two white cups) lined with seed papers to collect eggs from gravid females. The eggs were dried slowly in the insectary and kept for up to six months.

Mosquito colonies may adapt to our specific insectary and rearing regime. To control for that, in addition to the eight colonies in our lab (two La Lopé colonies, two Rabai forest colonies, and four Rabai domestic colonies, named “Powell” strains), we also acquired four more colonies from Rabai that had been reared in the McBride lab until the fifth generation (named ‘McBride’ strains). They were maintained as described in Rose et al. (2020). These four colonies consist of the copies of KBO1 and KBO2 that had been kept at the McBride lab, as well as two new peridomestic colonies (K65 from Chang’ombe village and K63 from Mbarekani) from Rabai. In total, we had two colonies from La Lopé and ten colonies from Rabai in our oviposition assays. All colonies were tested for their oviposition preferences for bacterial density and composition. In other experiments, we only tested the Rabai forest deep colony (“KBO1”, Powell strain) and the Kwa Bendegwa domestic colony, due to the limitations of time and number of mosquito eggs.

*Laboratory oviposition assays*

Each batch of the oviposition assays consisted of two to four colonies, with at least one forest colony and one village colony. We synchronized all colonies from hatching until the end of the experiment. The mosquitoes used for the laboratory oviposition assays were reared in a similar protocol as the main colonies described in the main texts, with a few changes to reduce between-experiment variations. Firstly, after hatching, the first-instar larvae were transferred into new larval trays with the density of one larva per 5mL water and fed with a fixed amount of larval food. Secondly, we kept around 360 adults, including both sexes, in a 17.5 cm cube cage (BugDorm-4M1515) instead of the larger cage for colony maintenance. Lastly, we fed females with sheep blood only once, about 5-8 days after more than 90% of pupae emerge into adults. Females were allowed to feed for one hour. We removed all males and females that did not appear engorged immediately after feeding.

Experiments started roughly 72 hours after blood-feeding. We used a two-choice design except for the experiment examining bacterial density, which used five choices. Specifically, five gravid females were transferred into a 15 x 15 x 15 cm customized cage with mesh covering both lateral and top sides (Figure S1). We used more than one animal per cage as preliminary trials with single females per cage rendered very low response rates. We placed two or five black cups (one-oz plastic food container cups, <https://www.amazon.com/gp/product/B018476QM6/ref=ppx_yo_dt_b_asin_title_o00_s00?ie=UTF8&psc=1> ) in the cage, each containing 12 mL solution that differs in the variable of interest. The cups were lined with seed-germination papers for collecting eggs. Positions of the cups with different choices were selected randomly among the two or five fixed cup positions (Figure S1). The experimental cages were kept in an environmental chamber (model PG031, Darwin Chambers Company, USA) with 27 ˚C, 70% humidity, 10 lux light intensity, and a 12h/12h light/dark cycle, which was more accurately regulated than the insectary room. Locations of the cages in the incubator were also assigned randomly. We allowed the females to lay eggs for 24 hours, and counted the number of eggs in each cup at the end of the experiments.

The specific conditions of the cups in each assay were informed by the field data to represent either forest or village larval sites (see Table S3 for details about each experiment). For the experiment that tested bacterial community composition specifically, we created forest and village type of bacterial community by inoculating water samples collected from forest and village larval sites in nutritionally rich Lysogeny broth (LB). After growing the two bacterial cultures overnight, we diluted them to the same cell density and used them as the two choices in the behavioral assays. Although the bacterial communities in these LB cultures likely varied from the actual bacterial communities in natural larval sites, they should still contain representative bacterial taxa from each habitat. In the experiment testing oviposition preference for bacterial density, we generated the bacteria solution similarly as described above, excepted that the bacterial culture were started with half forest water samples and half domestic water samples.

**Reference**

Gülden, E., Vudattu, N. K., Deng, S., Preston-Hurlburt, P., Mamula, M., Reed, J. C., Mohandas, S., Herold, B. C., Torres, R., & Vieira, S. M. (2017). Microbiota control immune regulation in humanized mice. *JCI insight,* **2**(21).

Kozich, J. J., Westcott, S. L., Baxter, N. T., Highlander, S. K., & Schloss, P. D. (2013). Development of a dual-index sequencing strategy and curation pipeline for analyzing amplicon sequence data on the MiSeq Illumina sequencing platform. *Applied and Environmental Microbiology,* **79**(17), 5112-5120. <https://doi.org/10.1128/AEM.01043-13>

Nelson, M. C., Morrison, H. G., Benjamino, J., Grim, S. L., & Graf, J. (2014). Analysis, optimization and verification of Illumina-generated 16S rRNA gene amplicon surveys. *PloS one,* **9**(4).

Rose, N. H., Sylla, M., Badolo, A., Lutomiah, J., Ayala, D., Aribodor, O. B., Ibe, N., Akorli, J., Otoo, S., Mutebi, J.-P., Kriete, A. L., Ewing, E. G., Sang, R., Gloria-Soria, A., Powell, J. R., Baker, R. E., White, B. J., Crawford, J. E., & McBride, C. S. (2020). Climate and Urbanization Drive Mosquito Preference for Humans. *Current Biology,* **30**(18), 3570-3579.e3576. <https://doi.org/10.1016/j.cub.2020.06.092>

Xia, S., Cosme, L. V., Lutomiah, J., Sang, R., Ngangue, M. F., Rahola, N., Ayala, D., & Powell, J. R. (2020). Genetic structure of the mosquito *Aedes aegypti* in local forest and domestic habitats in Gabon and Kenya. *Parasites & Vectors,* **13**(1), 417. <https://doi.org/10.1186/s13071-020-04278-w>

**Tables**

**Table S1.** Number of larval sites characterized for different groups of environmental variables

| Field site location | Habitat | *Aedes aegypti* | Physical characteristics | Microbial density | Bacteria composition | Volatile profile |
| --- | --- | --- | --- | --- | --- | --- |
| La Lopé, Gabon | Forest | Present | 5 | 5 | 5 | n.a.^*^ |
|  |  | Absent | 48 | 10 | 33 | n.a.^*^ |
|  | Peridomestic  (Village) | Present | 13 | 10 | 10 | n.a.^*^ |
|  |  | Absent | 24 | 12 | 23 | n.a.^*^ |
|  | **Total** |  | 90 | 37 | 71 | n.a.^*^ |
| Rabai, Kenya | Forest | Present | 15 | 15 | 15 | 7 |
|  |  | Absent | 22 | 11 | 22 | 12 |
|  | Peridomestic  (Village) | Present | 8 | 8 | 8 | 5 |
|  |  | Absent | 1 | 1 | 1 | 1 |
|  | Domestic  (Village) | Present | 22 | 22 | 22 | 17 |
|  | **Total** |  | 68 | 57 | 68 | 42 |

^*^ Volatile samples were not collected in La Lopé, Gabon.

**Table S2.** Method details of measuring physical variables of larval sites

| **Variable** | **Measurement method** | **Localities^*^** |
| --- | --- | --- |
| Longest diameter | We measured the longest diameter of the water surface using a measuring tape. For tires lying horizontally on the ground, this variable was measured as the diameter of the outer circle. | Both |
| Second diameter | We measured the longest diameter of the water surface that was perpendicular to the first diameter, using a measuring tape. For tires lying horizontal, this variable was measured as the diameter of the inner circle. | Both |
| Circumference | We calculated the circumference using the first and second diameters for containers with regular-shaped openings (e.g., round), or estimated it from photos. | Both |
| Surface area | We calculated the water surface area using the first and second diameters for containers with regular-shaped openings (e.g., round), or estimated it from photos. | Both |
| Container depth | We measured the distance from the bottom of the container to the lowest point of the container opening. | Rabai |
| Water depth | We measured the distance from the bottom of the container to the water surface | Rabai |
| Volume | If the container shape was regular (e.g., cylinder-shaped buckets and cans), the volume was calculated from the surface area and water depth. Otherwise, we estimated water volume by collecting all water from the larval site into a measuring bottle. When the measurement of the exact volume was not possible, the volume was estimated by researchers in the field. | Both |
| Height of the container opening | We measured the distance between the lowest point of the container opening to the ground next to the site. For rock pools and other breeding sites that sit directly on the ground, the height was zero. | Both |
| Temperature difference | Ambient temperature was measured by HOBO UX100-011 Temperature and Relative Humidity Loggers (Onset, MA, USA). A logger was placed beside the larval site until the read stabilized. Another logger calibrated to the first one was placed in the field station to record the diurnal fluctuation of ambient temperature. The temperature difference was calculated as the temperature measured by the larval sites minuses the temperature in the field station at the same time point. | Both |
| Humidity  difference | Relative humidity was measured by HOBO UX100-011 Temperature and Relative Humidity Loggers (Onset, MA, USA). A logger was placed beside the larval site until the read stabilized. Another logger calibrated to the first one was placed in the field station to record the diurnal fluctuation of relative humidity. The humidity difference was calculated as the relative humidity measured by the larval sites minuses the relative humidity in the field station at the same time point. | Both |
| Canopy  coverage | We measured canopy coverage by a spherical densiometer (Convex Model A, Forestry supply # 43887). The spherical densiometer was held as close to the opening of the larval site as possible. Canopy coverage was estimated facing north, west, south, and east, and we calculated their mean. Directions were determined by a compass in the forest. | Both |
| Water pH^+^ | In **La Lopé**, we measured water pH by a WTW-3110 pH-meter (Xylem, USA) from water samples collected in 50 mL sterile conical tubes less than 24 hours from collection. The water samples are kept in an icebox or at 4 degrees until returning to the field station, and then recovered to room temperature before measuring.  In **Rabai,** we measured water pH by a Hach Pocket Pro+ Multi 2 Tester (Hatch, USA) directly from the larval sites in the field. | Both |
| Conductivity^+^ | In **La Lopé**, we measured water conductivity by a WTW-3310 conductivity-meter (Xylem, USA) from water samples collected in 50 mL sterile conical tubes less than 24 hours from collection. The water samples are kept in an icebox or at 4 degrees until returning to the field station, and then recovered to room temperature before measuring.  In **Rabai,** we measured water conductivity by a Hach Pocket Pro+ Multi 2 Tester (Hatch, USA) directly from the larval sites in the field. | Both |
| Salinity | We measured water salinity by a Hach Pocket Pro+ Multi 2 Tester (Hatch, USA) directly from the larval sites in the field. | Rabai |
| Total dissolved solids (TDS) | We measured TDS by a Hach Pocket Pro+ Multi 2 Tester (Hatch, USA) directly from the larval sites in the field. | Rabai |
| Water temperature | We measured water temperature by a Hach Pocket Pro+ Multi 2 Tester (Hatch, USA) directly from the larval sites in the field. | Rabai |

^*^ This column indicates whether this variable was measured in both La Lopé and Rabai (‘Both’) or only in Rabai (‘Rabai’).

^+^ pH and conductivity were measured slightly differently in La Lopé and Rabai due to having different equipment.

**Table S3.** Details of laboratory oviposition assays

| **Variable** | **Methods to prepare oviposition choices** | **The choice resembling forest larval sites** | **The choice resembling village larval sites** | **Mosquito colonies** |
| --- | --- | --- | --- | --- |
| Water samples collected in the field | About 10 mL of water samples were collected from randomly selected 18 larval sites in Rabai forest and 18 in Rabai villages. The water samples were frozen at -20 ˚C. The forest and village water samples were randomly paired to create 18 pairs and used in an oviposition assay with nine cages of forest colonies and nine cages of domestic colonies. | Water samples were collected directly from Rabai forest larval sites, including sites present and absent of *Ae. aegypti*. | Water samples were collected directly from Rabai domestic larval sites, including sites present and absent of *Ae. aegypti*. | Kwa Bendegwa domestic colony, Powell strain;  Rabai forest deep colony(“KBO1”), Powell strain |
| pH | We adjusted the pH of 1x Phosphate-buffered saline (PBS) solution using hydrochloric acid (HCl) and sodium hydroxide (NaOH) to the desired value. | pH = 7.18 at the beginning of the experiment, which roughly equals the median pH of forest *Ae. aegypti* present larval sites in Rabai (pH = 7.2). The pH at the end of the experiment was 7.1. | pH = 8.71 at the beginning of the experiment, which is slightly higher than the median pH of domestic *Ae. aegypti* present larval sites in Rabai (pH = 8.4). The pH at the end of the experiment was 7.8. | Kwa Bendegwa domestic colony, Powell strain;  Rabai forest deep colony(“KBO1”), Powell strain |
| Shading | We placed each experimental cage in the center of a 17.8 x 17.8 x 17.8 cm cardboard box that allows light into the cage only from the top. The top side of the box was divided evenly into two halves, each covering one of the two cups in the cage. The two halves were modified to represent the shading of the forest and village larval sites in Rabai. To maximize the difference in shading, we placed the two oviposition cups against the opposite walls of the cage instead of 7.6 cm away as in other experiments. | The ‘forest’ half of the top side of the cardboard box has 30 holes of 0.8 cm diameter wide, which in total counts for ~ 8% of the surface area. This condition mimicked the median canopy coverage forest *Ae. aegypti* present larval sites in Rabai (92%). | The ‘domestic’ half of the top side of the cardboard box has no holes, mimicking the complete canopy coverage in most Rabai domestic larval sites. | Kwa Bendegwa domestic colony, Powell strain;  Rabai forest deep colony(“KBO1”), Powell strain |
| Combination of pH, salinity, and shading | We adjusted the pH of distilled water using hydrochloric acid (HCl) and sodium hydroxide (NaOH) and adjusted the conductivity using sodium chloride (NaCl). Different shading conditions were created as described above. | pH = 7.11, conductivity = 901 μL, shading = 92% | pH = 8.30, conductivity = 503 μL, shading = 100% | Kwa Bendegwa domestic colony, Powell strain;  Rabai forest deep colony(“KBO1”), Powell strain |
| Larval density | We hatched eggs of a Rabai forest colony and a Rabai domestic colony simultaneously. The second-instar larvae of each colony were transferred to two new trays with 800 mL distilled water at different larval densities (i.e., four larval trays in total: two colonies x two larval densities). We added 50 pallets of fish food per tray on the same day of larvae transferring and removed all larvae after three days. We then mixed the two trays of water with the same larval density in equal quantity and used them as the choices in the oviposition assay. | Water holding larvae for 3 days at the density of 50 larvae / 800 mL (1 larva / 16 mL). This larval density roughly matched the median larval density of all mosquito species in forest *Ae. aegypti* present larval sites in Rabai*.* | Water holding larvae for three days at a density of 1 larva / 800 mL. This larval density roughly matched the median larval density in domestic *Ae. aegypti* present larval sites in Rabai*.* | Kwa Bendegwa domestic colony, Powell strain;  Rabai forest deep colony(“KBO1”), Powell strain |
| Bacterial community composition ^*^ | During fieldwork, we preserved water samples from a subset of larval sites that were *Ae. aegypti* present using 80% glycerol. These samples included ten from La Lopé village, five from La Lopé forest, ten from Rabai forest, and ten from Rabai village. The glycerol preservation allowed the bacteria to stay alive with minimal changes over time. We kept the preservations at -80 ˚C.  To start a bacteria culture, we mixed glycerol preservations from each habitat in each locality with equal quantity to create four mixed “seed” stocks (La Lopé forest, La Lopé village, Rabai forest, Rabai village). We then inoculated the forest and village seed stock into two pre-sterilized 100 mL flasks, each containing 10 mL Lysogeny broth (LB). The bacterial cultures were shaken at 200 rpm at 37 ˚C for 24 hours. The cell densities of the two cultures were measured by OD600 light absorption. We first diluted the bacterial cultures to 1.25x10^9^ cells/mL in 10 mL LB media and then added 500 mL sterilized water. The final cell density is 2.5x10^7^ cells/mL. The diluted bacterial solutions made from the forest vs. village bacterial seed stocks were used as the two oviposition choices. | The diluted bacterial culture generated using bacterial glycerol samples collected from the forest *Ae. aegypti* present larval sites. | The diluted bacterial culture generated using bacterial glycerol samples collected from the village *Ae. aegypti* present larval sites. | All colonies |
| Bacterial density ^*^ | We generated bacterial cultures following the same protocol described above but inoculated the one LB media with both the forest and the village bacterial seed stocks. After the 24-hour growth, the bacterial culture was diluted using fresh LB to four different cell densities: 1.25x10^9^, 2.5x10^8^, 5x10^7^, and 1x10^7^ cells/mL, and then added to 500 mL sterilized water. The final cell densities were 2.5x10^7^, 5x10^6^, 1x10^6^, and 2x10^5^ cells/mL. A fifth choice was 10 mL LB in 500 mL sterilized water with no bacteria (0 cells/mL). | The median bacterial density in forest *Ae. aegypti* present larval sites in Rabai was 1.5x10^6^ cells/mL. | The median bacterial density in domestic *Ae. aegypti* present larval sites in Rabai was 3.0x10^5^ cells/mL. | All colonies |

^*^ We used the two La Lopé bacterial seed stocks in oviposition assays with the La Lopé *Ae. aegypti* colonies and the Rabai bacterial seed stocks in oviposition assays with the Rabai *Ae. aegypti* colonies.

**Table S4.** Comparison of microbial density and bacterial community alpha diversity in La Lopé

| Comparison^#^ | | | Variables | | | | |
| --- | --- | --- | --- | --- | --- | --- | --- |
|  |  |  | Microbial density^+^ | Shannon index  ASV level^+^ | Shannon index  Species level^+^ | Shannon index  Genus level^+^ | Shannon index  Family level^+^ |
| Forest | vs. | Peridomestic | W=138, p=0.412 | W=487, p=0.541 | **W=387, p=0.026** | **W=390, p=0.029** | W=411, p=0.062 |
| Forest **+** | vs. | Peridomestic **+** | W=26, p=1 | W=16, p=0.871 | W=11, p=0.397 | W=10, p=0.301 | W=11, p=0.397 |
| *Ae. aegypti* **+** | vs. | *Ae. aegypti* **-** | W=170, p=0.889 | W=508, p=0.871 | W=501, p=0.770 | W=493, p=0.914 | W=491, p=0.962 |
| Forest **+** | vs. | Forest **-** | W=33, p=1 | W=78, p=0.871 | W=92, p=0.770 | W=94, p=0.914 | W=94, p=0.962 |
| Peridomestic **+** | vs. | Peridomestic **-** | W=55, p=1 | W=86, p=0.871 | W=87, p=0.770 | W=88, p=0.914 | W=89, p=0.962 |

**^#^** ‘+’ and ‘-’ denote *Ae. aegypti* present and absent sites, respectively

^+^ Wilcoxon rank-sum test with Holm correction for multiple comparisons. Statistically significant results were marked **in** **bold**.

**Table S5.** Comparison of microbial density and bacterial community alpha diversity in Rabai

| Comparison^#^ | | | Variables | | | | |
| --- | --- | --- | --- | --- | --- | --- | --- |
|  |  |  | Microbial density^+^ | Shannon index  ASV level^+^ | Shannon index  Species level^+^ | Shannon index  Genus level^+^ | Shannon index  Family level^+^ |
| Forest | vs. | Peridomestic | W=113, p=1 | W=105, p=0.732 | W=160, p=1 | W=176, p=1 | W=199, p=1 |
| Forest | vs. | Domestic | **W=482.5, p<0.001** | W=369, p=1 | W=471, p=1 | W=509, p=0.785 | W=522, p=0.508 |
| Peridomestic | vs. | Domestic | W=157, p=0.062 | W=119, p=1 | W=119, p=1 | W=122, p=1 | W=121, p=1 |
| Forest **+** | vs. | Peridomestic **+** | W=64, p=1 | W=43, p=1 | W=42, p=1 | W=46, p=1 | W=52, p=1 |
| Forest **+** | vs. | Domestic **+** | **W=286.5, p=0.001** | W=143, p=1 | W=170, p=1 | W=183, p=1 | W=186, p=1 |
| Peridomestic **+** | vs. | Domestic **+** | W=135, p=0.135 | W=102, p=1 | W=109, p=1 | W=113, p=1 | W=113, p=1 |
| *Ae. aegypti* **+** | vs. | *Ae. aegypti* **-** | W=193, p=0.537 | W=496, p=1 | W=622, p=1 | W=650, p=0.697 | W=671, p=0.374 |
| Forest **+** | vs. | Forest **-** | W=94, p=1 | W=178, p=1 | W=214, p=1 | W=212, p=0.912 | W=207, p=1 |

**^#^** ‘+’ and ‘-’ denote *Ae. aegypti* present and absent sites, respectively

^+^ Wilcoxon rank-sum test with Holm correction for multiple comparisons. Statistically significant results were marked **in** **bold**.

**Table S6.** Bacterial families with significantly different abundance between forest and village peridomestic larval sites in La Lopé

| Class: Order | Family | Frequency in forest samples | Frequency in village samples | Proportion in forest samples | Proportion in village samples | log2 fold change^*^ | p-value^*^ |
| --- | --- | --- | --- | --- | --- | --- | --- |
| Betaproteobacteria:  Unknown | Unknown | 1 | 2 | 0.015 | 0.002 | -1.592 | 0.031 |
| Flavobacteriia:  Flavobacteriales | Flavobacteriaceae | 2 | 2 | 0.216 | 0 | -1.671 | 0.038 |
| Betaproteobacteria:  Burkholderiales | Unknown | 11 | 0 | 0.035 | 0 | -1.950 | 0.019 |
| Unknown:  Unknown | Unknown | 4 | 0 | 0.035 | 0 | -2.182 | < 0.001 |
| Betaproteobacteria:  Methylophilales | Methylophilaceae | 7 | 1 | 0.017 | 0 | -2.277 | 0.043 |
| Spartobacteria:  Unknown | Unknown | 7 | 0 | 0.001 | 0 | -2.335 | 0.002 |
| Armatimonadetes_gp5:  Unknown | Unknown | 4 | 0 | 0.001 | 0 | -3.045 | < 0.001 |
| Betaproteobacteria:  Burkholderiales | Burkholderiaceae | 27 | 4 | 0.069 | 0.027 | -3.212 | 0.003 |
| Cyanobacteria:  Family_XIII | GpXIII | 1 | 0 | 0.002 | 0 | -3.261 | 0.049 |
| Gammaproteobacteria:  Methylococcales | Methylococcaceae | 1 | 0 | 0.014 | 0 | -3.345 | 0.022 |
| Gammaproteobacteria:  Aeromonadales | Aeromonadaceae | 9 | 0 | 0.002 | 0 | -3.389 | 0.004 |
| Chlamydiia:  Chlamydiales | Parachlamydiaceae | 1 | 0 | 0.025 | 0 | -3.530 | < 0.001 |
| Deltaproteobacteria:  Bdellovibrionales | Bacteriovoracaceae | 5 | 0 | 0.019 | 0 | -3.562 | 0.002 |
| Chlamydiia:  Chlamydiales | Simkaniaceae | 4 | 0 | 0.007 | 0 | -3.672 | 0.004 |
| Cyanobacteria:  Unknown | Unknown | 1 | 0 | 0.016 | 0 | -3.709 | 0.005 |
| Cyanobacteria:  Family_IX | GpIX | 5 | 0 | 0.002 | 0 | -4.646 | 0.002 |
| Holophagae:  Holophagales | Holophagaceae | 8 | 0 | 0.002 | 0 | -4.737 | < 0.001 |
| Actinobacteria:  Actinomycetales | Microbacteriaceae | 14 | 1 | 0.068 | 0.001 | -4.976 | < 0.001 |
| Cyanobacteria:  Family_XI | GpXI | 1 | 0 | 0.232 | 0 | -5.853 | 0.004 |
| Bacilli:  Bacillales | Bacillales_Incertae_Sedis_XII | 0 | 4 | 0 | 0.048 | 23.199 | < 0.001 |
| Alphaproteobacteria:  Rhodospirillales | Reyranella | 0 | 1 | 0 | 0.221 | 5.351 | < 0.001 |
| Actinobacteria:  Actinomycetales | Nocardiaceae | 0 | 3 | 0 | 0.011 | 4.502 | < 0.001 |
| Verrucomicrobiae:  Verrucomicrobiales | Verrucomicrobiaceae | 0 | 2 | 0 | 0.024 | 3.585 | < 0.001 |
| Oligoflexia:  Oligoflexales | Oligoflexaceae | 1 | 9 | 0.001 | 0.002 | 3.459 | 0.004 |
| Acidobacteria_Gp4:  Aridibacter | Unknown | 1 | 4 | 0 | 0.005 | 3.442 | 0.019 |
| Acidobacteria_Gp3:  Gp3 | Unknown | 0 | 8 | 0 | 0.003 | 3.255 | < 0.001 |
| Actinobacteria:  Actinomycetales | Geodermatophilaceae | 0 | 6 | 0 | 0.002 | 3.231 | 0.026 |
| Alphaproteobacteria:  Rhizobiales | Xanthobacteraceae | 0 | 7 | 0 | 0.028 | 2.776 | 0.005 |
| Alphaproteobacteria:  Caulobacterales | Caulobacteraceae | 10 | 20 | 0.002 | 0.007 | 2.268 | < 0.001 |
| Gammaproteobacteria:  Pseudomonadales | Pseudomonadaceae | 0 | 1 | 0 | 0.085 | 2.188 | 0.037 |
| Betaproteobacteria:  Burkholderiales | Oxalobacteraceae | 0 | 19 | 0 | 0.071 | 2.041 | 0.007 |
| Alphaproteobacteria:  Rhizobiales | Methylobacteriaceae | 10 | 21 | 0.001 | 0.002 | 2.002 | 0.001 |
| Sphingobacteriia:  Sphingobacteriales | Sphingobacteriaceae | 0 | 2 | 0 | 0.030 | 1.944 | 0.019 |
| Alphaproteobacteria:  Rhodospirillales | Acetobacteraceae | 0 | 7 | 0 | 0.022 | 1.163 | 0.049 |
| Epsilonproteobacteria:  Campylobacterales | Campylobacteraceae | 0 | 1 | 0 | 0.056 | -4.509 | 0.019 |

^*^ Calculate by R package *DESeq2.*

**Table S7.** Bacterial families with significantly different abundance between forest and village (domestic, peridomestic) larval sites in Rabai

| Class: Order | Family | Frequency in forest samples | Frequency in village samples | Proportion in forest samples | Proportion in village samples | log2 fold change^*^ | p-value^*^ |
| --- | --- | --- | --- | --- | --- | --- | --- |
| Deltaproteobacteria:  Desulfuromonadales | Geobacteraceae | 1 | 0 | 0.248 | 0 | 6.559 | < 0.001 |
| Betaproteobacteria:  Rhodocyclales | Rhodocyclaceae | 2 | 0 | 0.180 | 0 | 5.855 | < 0.001 |
| Bacilli:  Bacillales | Paenibacillaceae_1 | 12 | 0 | 0.003 | 0 | 5.512 | < 0.001 |
| Methanobacteria:  Methanobacteriales | Methanobacteriaceae | 4 | 0 | 0.001 | 0 | 5.378 | < 0.001 |
| Deltaproteobacteria:  Desulfobacterales | Desulfobulbaceae | 2 | 0 | 0.002 | 0 | 5.187 | 0.001 |
| Clostridia:  Clostridiales | Peptococcaceae_1 | 1 | 0 | 0.008 | 0 | 4.987 | < 0.001 |
| Alphaproteobacteria:  Rhizobiales | Methylocystaceae | 3 | 0 | 0.026 | 0 | 4.970 | < 0.001 |
| Clostridia:  Unknown | Unknown | 2 | 0 | 0.001 | 0 | 4.546 | 0.001 |
| Actinobacteria:  Coriobacteriales | Coriobacteriaceae | 3 | 0 | 0.002 | 0 | 4.161 | 0.019 |
| Actinobacteria:  Actinomycetales | Cellulomonadaceae | 7 | 0 | 0.015 | 0 | 4.136 | 0.004 |
| Clostridia:  Clostridiales | Unknown | 5 | 0 | 0 | 0 | 4.080 | < 0.001 |
| Deltaproteobacteria:  Desulfovibrionales | Desulfovibrionaceae | 4 | 0 | 0 | 0 | 4.017 | 0.007 |
| Bacilli:  Lactobacillales | Streptococcaceae | 7 | 3 | 0.021 | 0.003 | 3.689 | 0.044 |
| Gammaproteobacteria:  Pseudomonadales | Pseudomonadaceae | 15 | 7 | 0.034 | 0.005 | 3.637 | < 0.001 |
| Clostridia:  Clostridiales | Ruminococcaceae | 4 | 0 | 0.001 | 0 | 3.470 | < 0.001 |
| Clostridia:  Clostridiales | Clostridiales_Incertae_Sedis_XIII | 2 | 0 | 0 | 0 | 3.401 | 0.020 |
| Clostridia:  Clostridiales | Heliobacteriaceae | 1 | 0 | 0.001 | 0 | 3.248 | 0.002 |
| Bacteroidia:  Bacteroidales | Unknown | 1 | 0 | 0.001 | 0 | 3.173 | 0.039 |
| Gammaproteobacteria:  Enterobacteriales | Enterobacteriaceae | 10 | 2 | 0.043 | 0.001 | 3.095 | 0.003 |
| Actinobacteria:  Actinomycetales | Thermomonosporaceae | 1 | 0 | 0.001 | 0 | 2.946 | 0.035 |
| Bacilli:  Bacillales | Unknown | 30 | 16 | 0.008 | 0.002 | 2.717 | 0.001 |
| Actinobacteria:  Actinomycetales | Streptomycetaceae | 20 | 1 | 0.007 | 0 | 2.501 | 0.002 |
| Negativicutes:  Selenomonadales | Veillonellaceae | 3 | 0 | 0.001 | 0 | 2.412 | 0.008 |
| Bacilli:  Bacillales | Planococcaceae | 17 | 7 | 0.020 | 0.002 | 2.347 | 0.023 |
| Gammaproteobacteria:  Xanthomonadales | Xanthomonadaceae | 19 | 16 | 0.025 | 0.002 | 2.121 | 0.003 |
| Clostridia:  Clostridiales | Clostridiaceae_1 | 7 | 1 | 0.002 | 0 | 1.758 | 0.020 |
| Unknown:  Unknown | Unknown | 1 | 0 | 0.011 | 0 | -1.526 | 0.033 |
| Bacteroidia:  Bacteroidales | Porphyromonadaceae | 0 | 2 | 0 | 0.011 | 2.979 | 0.010 |
| Planctomycetia:  Planctomycetales | Planctomycetaceae | 0 | 2 | 0 | 0.010 | -1.584 | 0.030 |
| Verrucomicrobiae:  Verrucomicrobiales | Verrucomicrobiaceae | 2 | 2 | 0 | 0.005 | -1.844 | 0.032 |
| Alphaproteobacteria:  Rhizobiales | Methylobacteriaceae | 3 | 6 | 0 | 0.012 | -2.102 | 0.030 |
| Deltaproteobacteria:  Bdellovibrionales | Bdellovibrionaceae | 0 | 2 | 0 | 0.003 | -2.132 | 0.023 |
| Actinobacteria:  Actinomycetales | Micrococcaceae | 3 | 10 | 0 | 0.002 | -2.320 | 0.008 |
| Alphaproteobacteria:  Rhodospirillales | Acetobacteraceae | 0 | 4 | 0 | 0.018 | -2.554 | 0.001 |
| Alphaproteobacteria:  Rhodobacterales | Rhodobacteraceae | 0 | 12 | 0 | 0.027 | -2.666 | < 0.001 |
| Betaproteobacteria:  Unknown | Unknown | 0 | 6 | 0 | 0.007 | -2.993 | 0.030 |
| Cytophagia:  Cytophagales | Unknown | 0 | 3 | 0 | 0.001 | -3.327 | 0.023 |
| Gammaproteobacteria:  Legionellales | Legionellaceae | 0 | 1 | 0 | 0.014 | -3.568 | < 0.001 |
| Cytophagia:  Cytophagales | Cytophagaceae | 0 | 6 | 0 | 0.027 | -3.677 | < 0.001 |
| Unknown:  Unknown | Unknown | 0 | 12 | 0 | 0.005 | -3.708 | < 0.001 |
| Flavobacteriia:  Flavobacteriales | Cryomorphaceae | 0 | 3 | 0 | 0.007 | -3.833 | 0.008 |
| Chloroplast:  Chloroplast | Bacillariophyta | 0 | 5 | 0 | 0.002 | -5.268 | 0.005 |
| Bacilli:  Bacillales | Bacillales_Incertae_Sedis_XII | 2 | 20 | 0.001 | 0.022 | -5.770 | < 0.001 |
| Cytophagia:  Cytophagales | Cyclobacteriaceae | 0 | 10 | 0 | 0.006 | -5.853 | 0.001 |
| Betaproteobacteria:  Methylophilales | Methylophilaceae | 0 | 10 | 0 | 0.006 | -6.034 | < 0.001 |
| Deinococci:  Deinococcales | Deinococcaceae | 0 | 18 | 0 | 0.001 | -6.269 | < 0.001 |
| Alphaproteobacteria:  Rhodospirillales | Reyranella | 0 | 9 | 0 | 0.004 | -6.701 | < 0.001 |

^*^ Calculate by R package *DESeq2.*

**Table S8.** Confusion matrix of larval site classification in La Lopé by random forest

|  | Forest **+** | Forest **-** | Peridomestic **+** | Peridomestic **-** | Total |
| --- | --- | --- | --- | --- | --- |
| Forest **+** | **0** | 5 | 0 | 0 | 5 |
| Forest **-** | 0 | **28** | 0 | 0 | 28 |
| Peridomestic **+** | 0 | 0 | **2** | 8 | 10 |
| Peridomestic **-** | 0 | 4 | 3 | **13** | 20 |

The row names indicate the actual larval site groups, and the column names indicate the assigned larval site groups by the random forest model. “+” and “-” denote *Ae. aegypti* present and absent sites, respectively. The numbers of larval sites assigned to the correct groups are marked in bold.

**Table S9.** Confusion matrix of larval site classification in Rabai by random forest

|  | Forest **+** | Forest **-** | Peridomestic **+** | Peridomestic **-** | Domestic **+** | Total |
| --- | --- | --- | --- | --- | --- | --- |
| Forest **+** | **8** | 5 | 0 | 0 | 0 | 13 |
| Forest **-** | 7 | **3** | 0 | 0 | 0 | 10 |
| Peridomestic **+** | 0 | 0 | **4** | 0 | 4 | 8 |
| Peridomestic **-** | 0 | 0 | 1 | **0** | 0 | 1 |
| Domestic **+** | 0 | 0 | 1 | 0 | **19** | 20 |

Note: the row names indicate the actual larval site groups, and the column names indicate the assigned larval site groups by the random forest model. “+” and “-” denote *Ae. aegypti* present and absent sites, respectively. The numbers of larval sites assigned to the correct groups are marked in bold.

**Table S10.** Results of beta-binomial models and negative-binomial models for laboratory oviposition assays

| Variables | Colonies | Models^*^ | Model comparison results |
| --- | --- | --- | --- |
| Water samples from the field | Kwa Bendegwa domestic  & Rabai forest deep | Full model: egg counts in two cups ~ **Colony**  Null model: egg counts in two cups ~ 1 | Full model AIC: 226.69  Null model AIC: 225.76  ꭓ^2^ = 1.08, df = 1, p = 0.300 |
| Water pH |  |  | Full model AIC: 162.55  Null model AIC: 161.07  ꭓ^2^ = 0.52, df = 1, p = 0.472 |
| Shading |  |  | Full model AIC: 340.11  Null model AIC: 38.68  ꭓ^2^ = 0.58, df = 1, p = 0.447 |
| Combination of pH, conductivity, and shading |  |  | Full model AIC: 271.42  Null model AIC: 269.93  ꭓ^2^ = 0.52, df = 1, p = 0.472 |
| Larval density | Kwa Bendegwa domestic  & Rabai forest deep | Full model: egg counts in two cups ~ **Colony** +  (1\|Experiment ID)^#^  Null model: egg counts in two cups ~ 1 +  (1\|Experiment ID) | Full model AIC: 566.40  Null model AIC: 567.39  ꭓ^2^ = 2.98, df = 1, p = 0.084 |
| Bacterial community | La Lopé forest  &  La Lopé village | Full model: egg counts in two cups ~ **Colony**  Null model: egg counts in two cups ~ 1 | Full model AIC: 156.60  Null model AIC: 155.19  ꭓ^2^ = 0.59, df = 1, p = 0.44 |
|  | All Rabai colonies | Full model: egg counts in two cups ~ **Habitat** +  (1\| Colony) + (1\|Experiment ID)^##^  Null model: egg counts in two cups ~ 1 +  (1\| Colony) + (1\|Experiment ID) | Full model AIC: 816.87  Null model AIC: 814.46  ꭓ^2^ = 0.55, df = 1, p = 0.451 |
|  |  | Full model: egg counts in two cups ~ **Colony** +  (1\|Experiment ID)  Null model: egg counts in two cups ~ 1 +  (1\|Experiment ID) | Full model AIC: 820.15  Null model AIC: 810.46  ꭓ^2^ = 8.32, df = 9, p = 0.503 |
| Bacterial  density | La Lopé forest  &  La Lopé village | Full model: egg count of each cup ~  **Colony x Oviposition choice** +  (1\| Cage ID)  Null model: egg count of each cup ~  **Colony + Oviposition choice** +  (1\| Cage ID) | Full model AIC: 1039.6  Null model AIC: 1033.9  ꭓ^2^ = 2.29, df = 4, p = 0.683 |
|  | All Rabai colonies | Full model: egg count of each cup ~  **Habitat x Oviposition choice** +  (1\| Colony) + (1\|Experiment ID) + (1\| Cage ID) ^###^  Null model: egg count of each cup ~  **Habitat + Oviposition choice** +  (1\| Colony) + (1\|Experiment ID) + (1\| Cage ID) | Full model AIC: 4386  Null model AIC: 4374  ꭓ^2^ = 3.98, df = 8, p = 0.858 |
|  |  | Full model: egg count of each cup ~  **Colony x Oviposition choice** +  (1\|Experiment ID) + (1\| Cage ID)  Null model: egg count of each cup ~  **Colony + Oviposition choice** +  (1\|Experiment ID) + (1\| Cage ID) | Full model AIC: 4416.8  Null model AIC: 4370.7  ꭓ^2^ = 25.9, df = 36, p = 0.893 |

^*^ Negative-binomial models for testing bacterial density and beta-binomial models for the rest of the oviposition assays.

^#^ For oviposition assays that were conducted in multiple experiment batches, experiment ID was included as a random effect.

^##^ Colonies were included as random factors in statistical models examining the effects of habitats.

^###^ Cage ID was included in oviposition assays to control for the paired structure of the five egg counts in each cage.

**Figures**


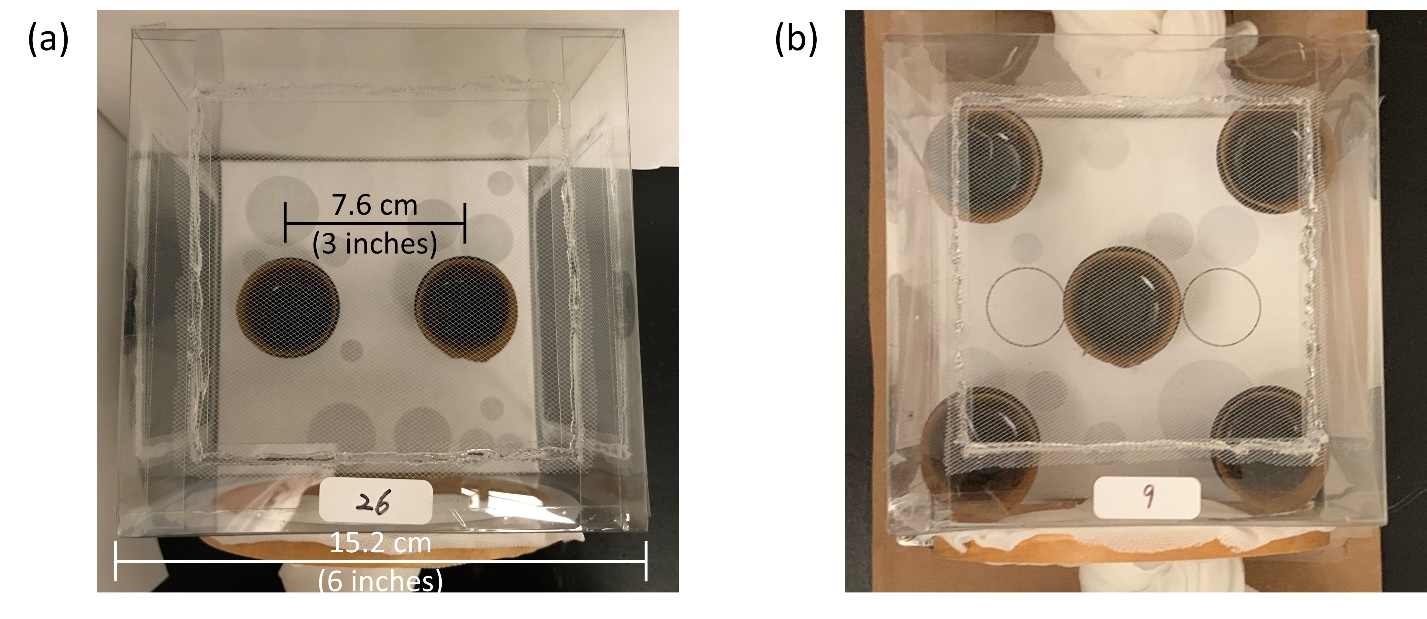


**Figure S1.** Designs of the laboratory oviposition assays with (a) two choices for testing most variables and (b) five choices for testing bacterial density. The cage was built from a 15.2 x 15.2 x 15.2 cm transparent plastic box with fine meshes covering the top and the two lateral sides. A cloth sleeve is attached to the front side’s opening (bottom in the photos) through which the mosquitoes were introduced. The cage has a white bottom with randomly generated gray circles that provide visual stimuli for the mosquitoes to navigate in the cage. The black cups, each lined with a piece of 4 x 13 cm seed germination paper, were 7.6 cm away from each other in the two-choice cages (a). In the five-choice experiments (b), the five cups were located at the four corners of the cage and the center. In each cage, the different oviposition choices were randomly assigned to the two or five cup positions.


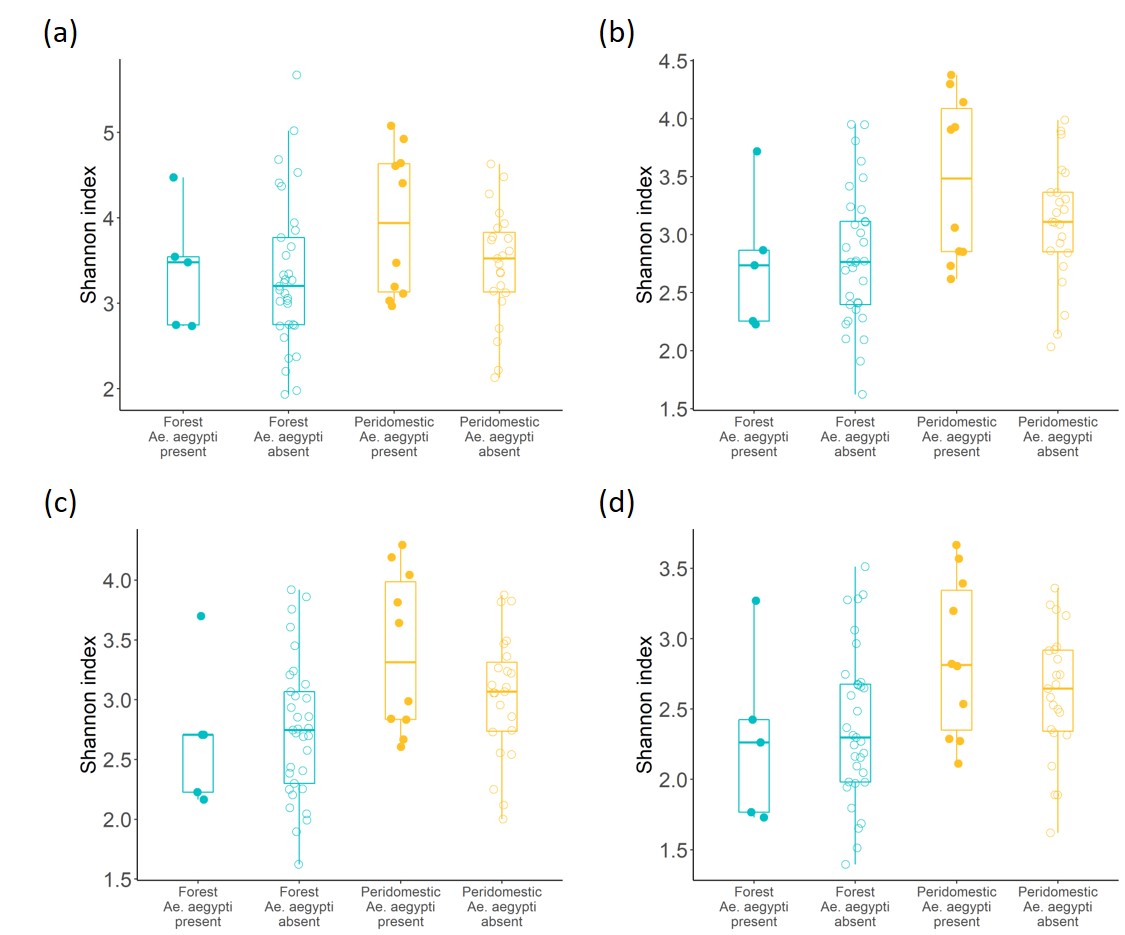


**Figure S2.** Comparison of the Shannon index of bacterial community in La Lopé larval sites at different taxonomic levels: (a) ASV, (b) Species, (c) Genus, and (d) Family. Each point represents a single larval site, and the boxplots show the minimum, 25% quartile, median, 75% quartile, and maximum of all values. The colors and shapes are as in Figure 2 in the main document. Differences between groups were tested using pairwise Wilcoxon rank-sum tests with Holm multiple comparison corrections. No significant difference was found in any tests (Table S4).


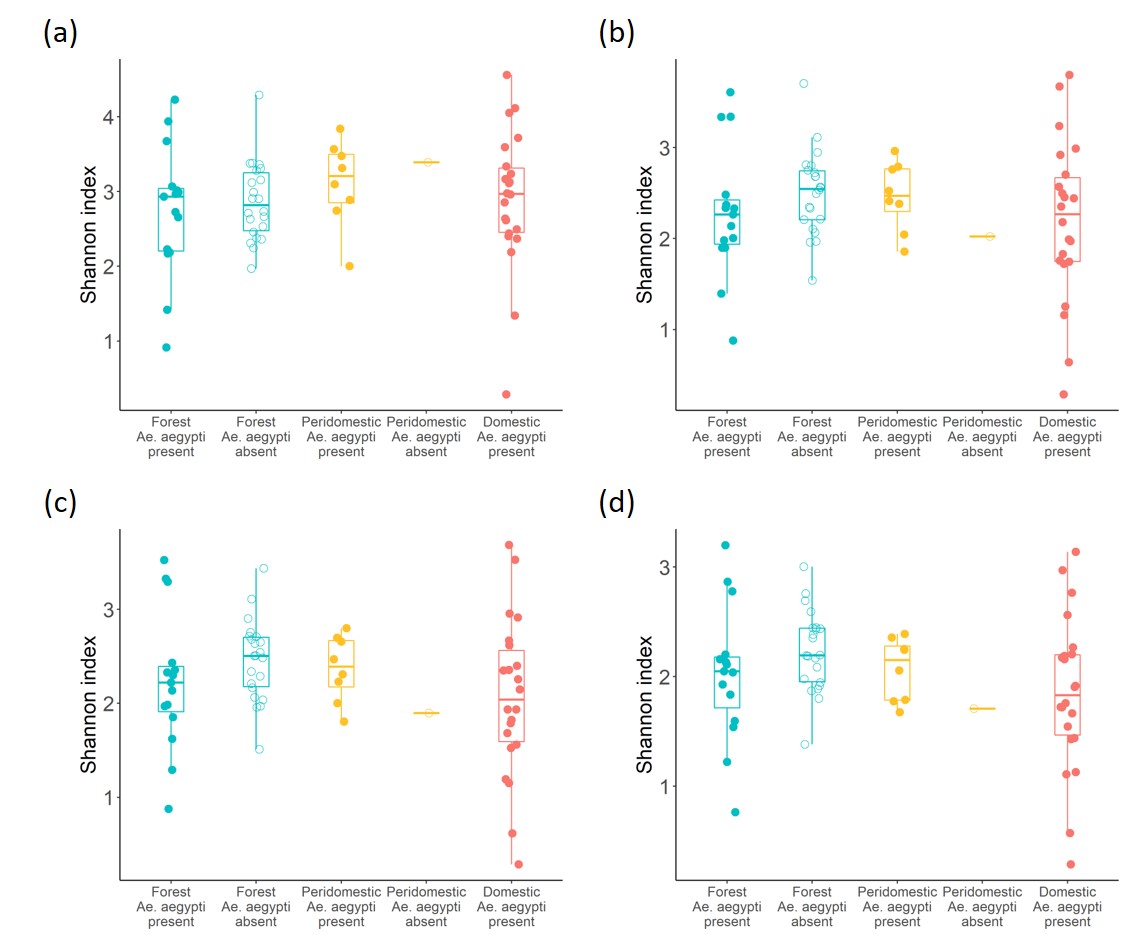


**Figure S3.** Comparison of the Shannon index of bacterial community in Rabai larval sites at different taxonomic levels: (a) ASV, (b) Species, (c) Genus, and (d) Family. Each point represents a single larval site, and the boxplots show the minimum, 25% quartile, median, 75% quartile, and maximum of all values. The colors and shapes are as in Figure 2 in the main document. Differences between groups were tested using pairwise Wilcoxon rank-sum tests with Holm multiple comparison corrections. No significant difference was found in any tests (Table S5).


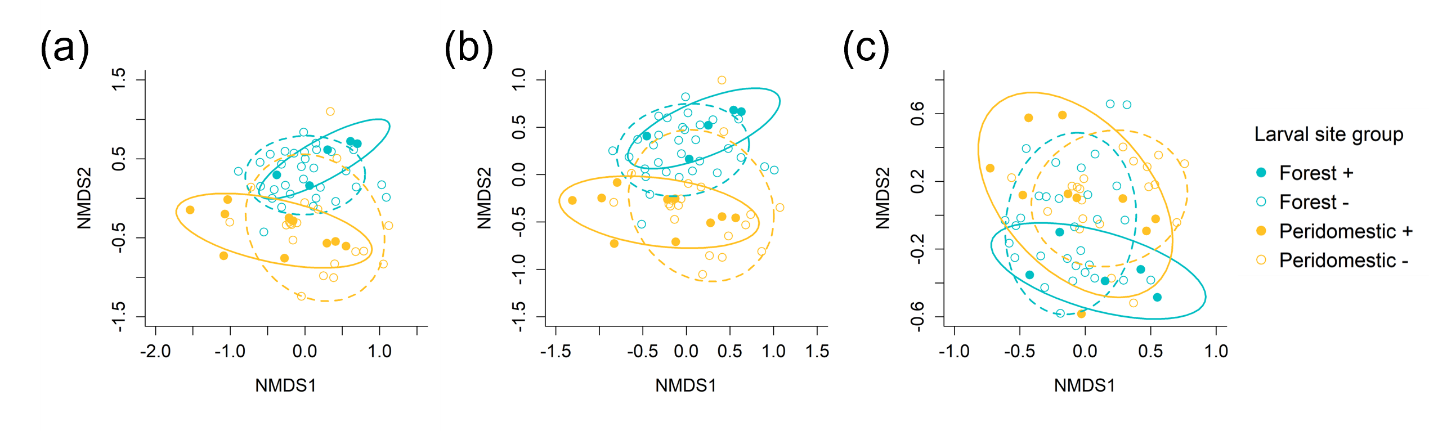


**Figure S4.** Non-metric multidimensional scaling (NMDS) analysis of bacterial community compositions in La Lopé larval site at (a) Species, (b) Genus, and (c) Family level. Each point represents a larval site. The color and shape of points and ellipses are the same as in Figure 2 in the main document.


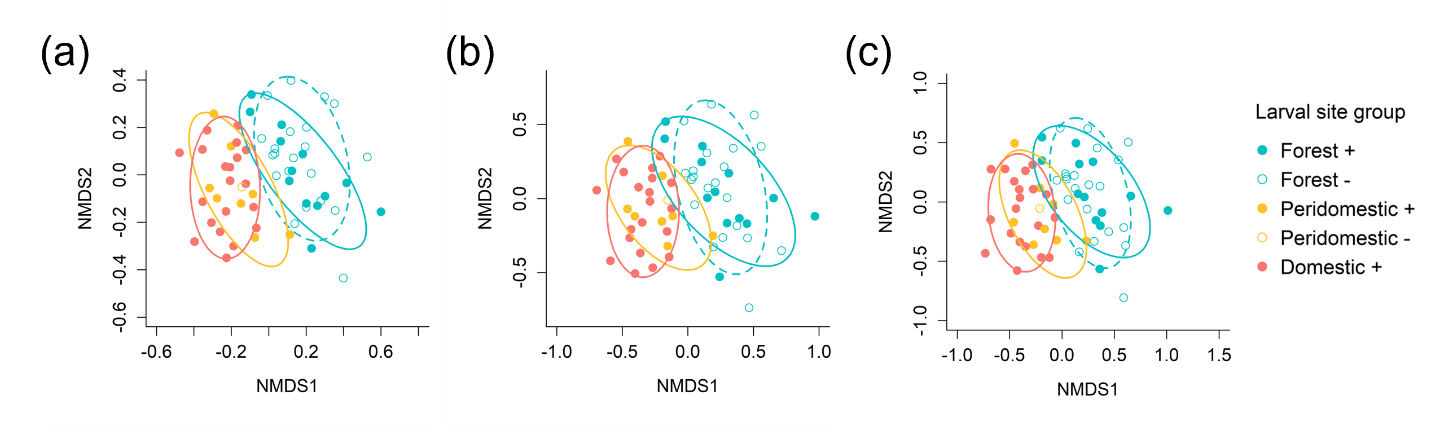


**Figure S5.** Non-metric multidimensional scaling (NMDS) analysis of bacterial community compositions in Rabai larval site at (a) Species, (b) Genus, and (c) Family level. Each point represents a larval site. The color and shape of points and ellipses are the same as in Figure 2 in the main document.


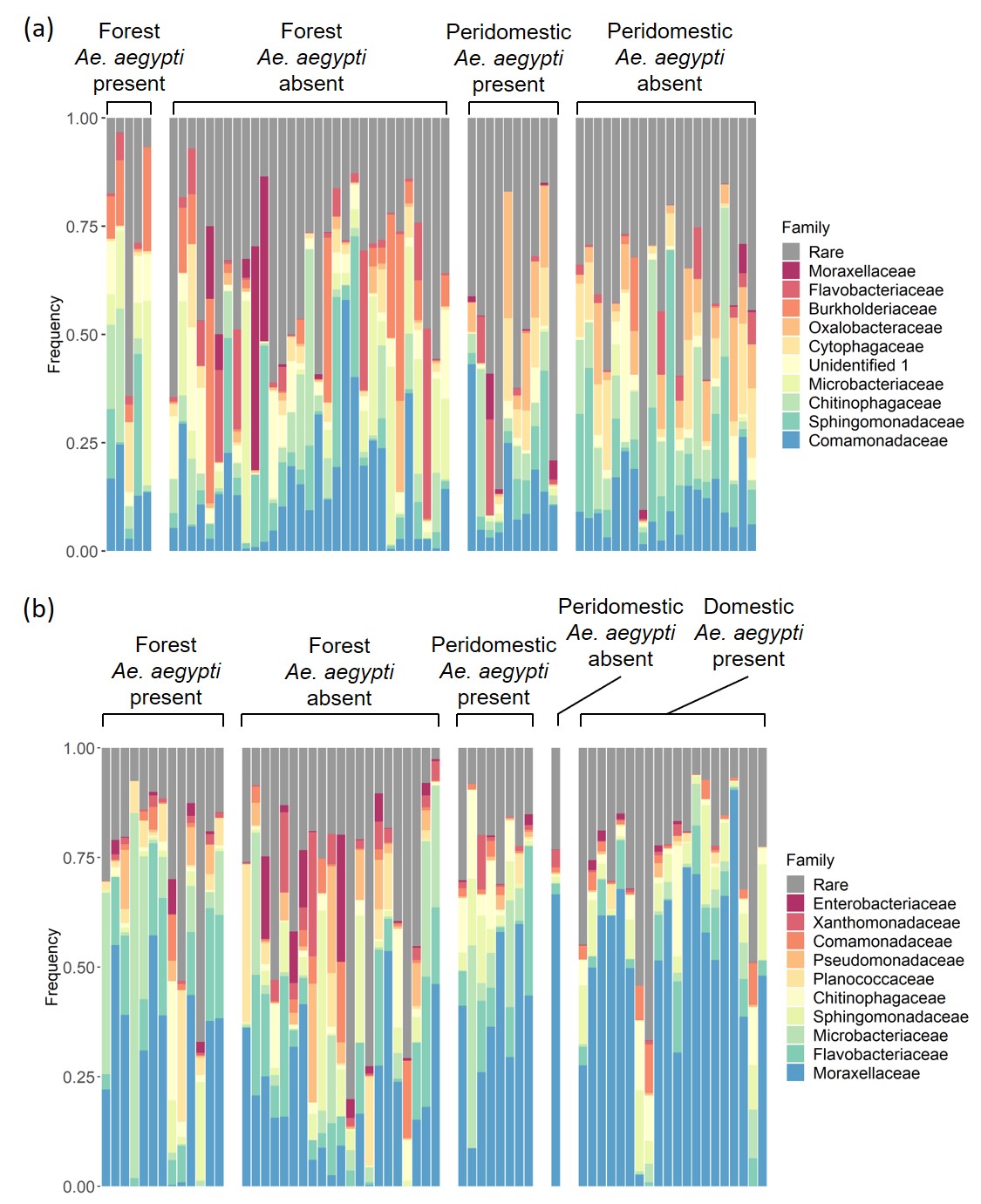


**Figure S6.** Frequency of the top ten bacterial families in larval sites in (a) La Lopé and (b) Rabai. Each bar represents a larval site, and the length of each color represents the proportion of the corresponding family in the site. Other bacterial families are grouped in the ‘Rare’ category, which shows as gray in the bar plots.


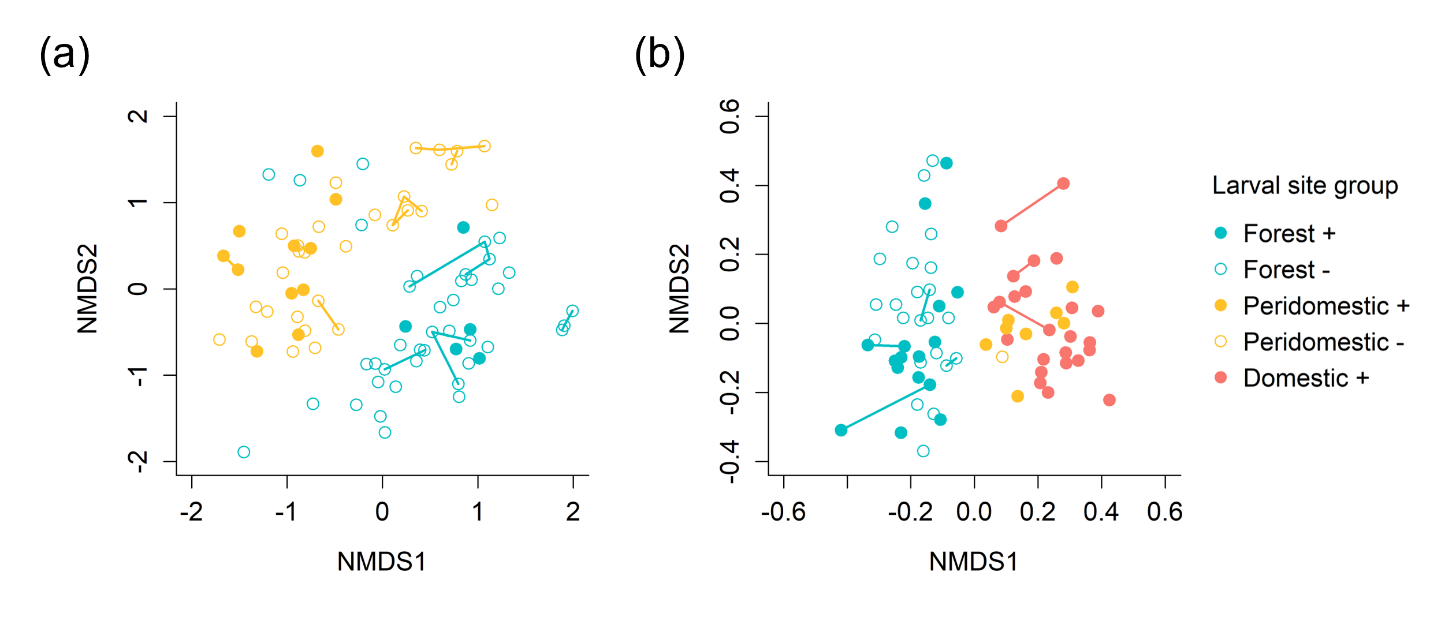


**Figure S7.** Temporal variations of bacterial community compositions at the ASV level in (a) La Lopé and (b) Rabai. Each point represents a larval site. The color and shape of points and ellipses are the same as in Figure 2 in the main document. Bacterial samples collected from the same larval sites at different times are linked with segments.


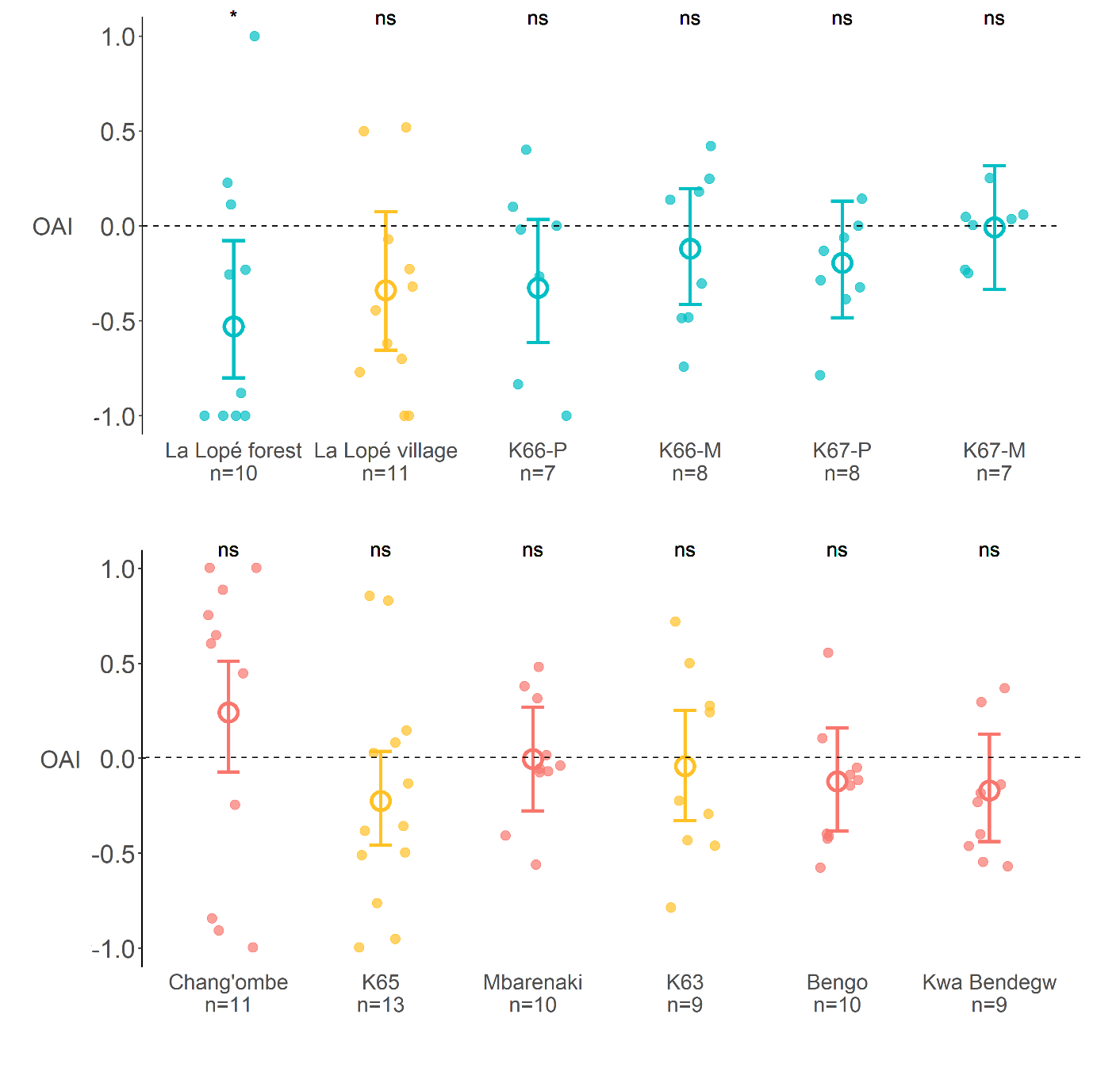


**Figure S8.** Colony-wise results of the laboratory oviposition assays on forest versus village bacteria culture (Figure 4f in the main document). Each point represents one cage with five gravid females, and the color indicates the habitat where the colony originated. Higher OAI indicates a preference for the forest bacteria cultures. “-P” and “-M” represent the two copies of the same colony maintained at the Powell lab at Yale University and the McBride lab at Princeton University. A beta-binomial model was used to test differential preference among colonies, which resulted in no significant colony effects (Table S10). The model also estimated the mean OAIs and the 95% confidence intervals indicated by the open cycles and the error bars. The asterisks and ‘ns’ (not significant) indicate whether the 95% CI excludes zero.


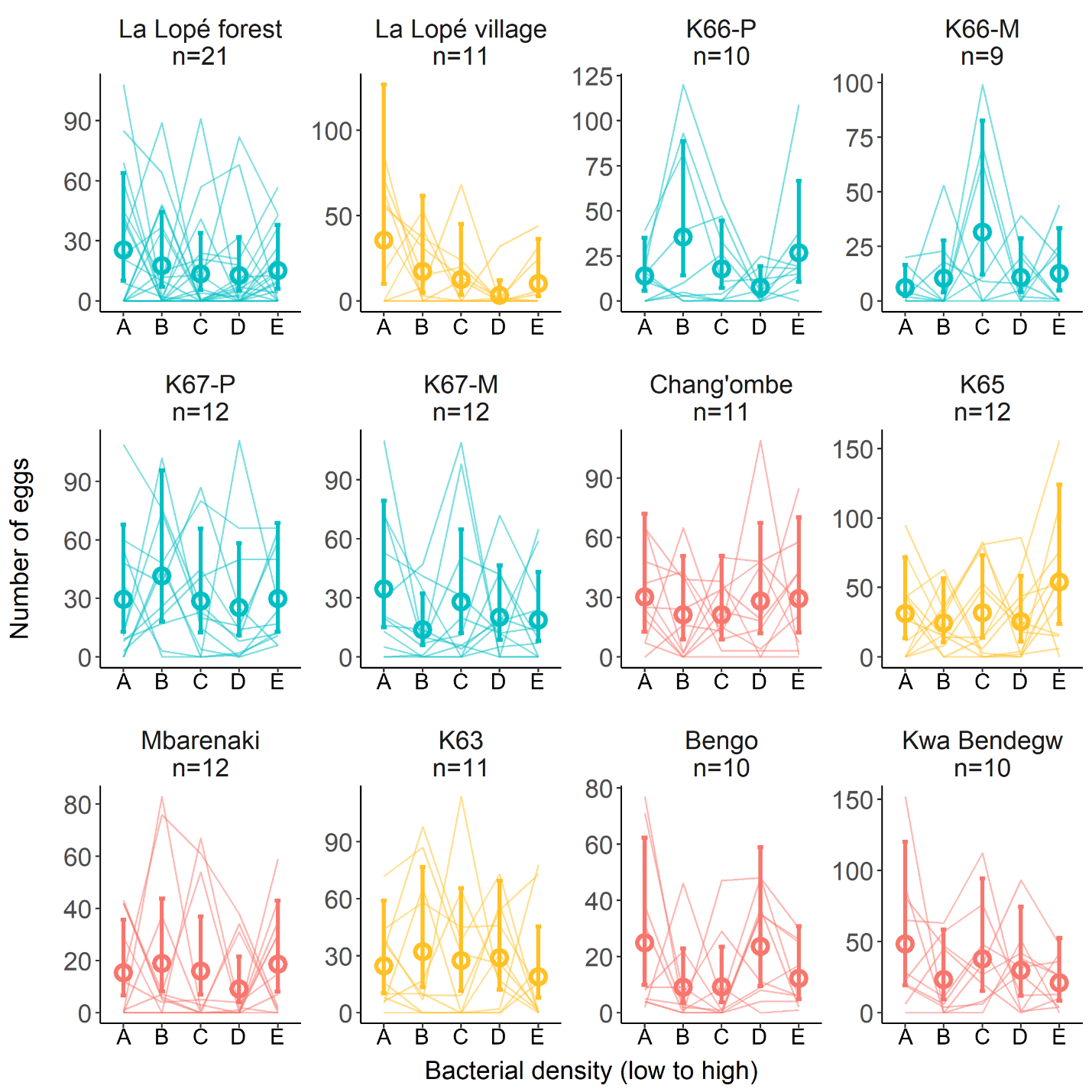


**Figure S9.** Colony-wise results of the laboratory oviposition assay on bacterial density. Five cups were provided in each cage with increasing bacterial density at (A) 0, (B) 2x10^5^, (C) 1x10^6^, (D) 5x10^6^, € 2.5x10^7^ cells/mL (details in Table S3). Each line connects the five egg counts in one cage. Colors represent the habitats of the colonies. A negative binomial model estimated the mean number of eggs in each bacterial density and a 95% confidence interval, indicated by the open cycles and the error bars.
